# The Status of Ethnobotanical Knowledge of Medicinal Plants and the Impacts of Resettlement in Delanta, Northwestern Wello, Northern Ethiopia

**DOI:** 10.1155/2016/5060247

**Published:** 2016-01-13

**Authors:** Misganaw Meragiaw, Zemede Asfaw, Mekuria Argaw

**Affiliations:** ^1^Department of Plant Biology & Biodiversity Management, College of Natural Sciences, National Herbarium, Addis Ababa University, P.O. Box 3434, Addis Ababa, Ethiopia; ^2^Center for Environmental Sciences, College of Natural Sciences, Addis Ababa University, P.O. Box 1176, Addis Ababa, Ethiopia

## Abstract

The present study was conducted in Delanta (Ethiopia) to examine the use of medicinal plants and investigate the impacts of the 1984/85 resettlement program on the local people's knowledge on herbal medicine and its uses. The research was conducted with 72 informants in six study sites through semistructured interviews, group discussion, and market survey. In this study, 133 species belonging to 116 genera and 57 families were documented. These plants were mentioned for uses in the treatment of about 76 human and livestock ailments. The family Asteraceae was represented by the highest number with 14 species. Herbs accounted for 52.6% of the total species and leaves (32.6%) were the most frequently used parts. The analysis showed that the resettlement program has both positive and negative impacts on nature rehabilitation and local knowledge along with many human induced threats. Most of the plant knowledge is held by traditional healers and permanent residents. The people's preference for some medicinal plants gave indications of continuity of the ethnomedicinal information among the inhabitants. The findings inform that efforts need to be directed to in situ conservation in two of the plant community types which could protect a good proportion (about 50%) of the medicinal plant species.

## 1. Introduction

The concept of ethnobotanical knowledge has originated from local people, which has the potential to redress some of the shortcomings of contemporary Western knowledge [1, 2]. It is passed down from generation to generation and closely interwoven with people's cultural values [3]. Traditional societies throughout the world hold a wealth of such knowledge which they have built up during prolonged interactions with the natural world and which remains fundamental to their physical, spiritual, and social interests [4, 5]. While plants can provide multiple uses, the traditional curative practice of health problem is among the most important ones for peoples' lives [6–8] and it is also one of the sources of modern health treatment [9]. Since time immemorial, traditional medicinal plants (TMPs) have been used in virtually all cultures for treatment of most human and livestock ailments [3, 10]. The uses of plant species as TMPs represent by far the biggest human use in terms of number of species of the natural world [11]. It is estimated that 70–80% of people worldwide [12] and 80% of the people of Ethiopia rely chiefly on traditional herbal medicines to meet their primary healthcare needs. The ways to combat diseases through TMPs are also as diverse as the different cultures [13–15]. The natural plant world is thus full of potential for new drug discovery. There are undoubtedly many more secrets still hidden in the world of plants [16]. These resources are found in locally available plants and they benefit from local knowledge (LK) that is simple to use and affordable. Reasonable support for TMPs will not only help bridge some of the gaps between the demand for and supply of modern pharmaceuticals, but also widen healthcare alternatives for posterity [4, 15].

The most serious dilemma facing Delanta was recurrent droughts [17, 18] that often led to famines being usually accompanied by epidemics of different diseases [19]. Resettlement of local communities, acculturation, and inadequate understanding of the potentials of TMPs and the associated LK to the present and future generations have led to a decline in sustainable use of these biological resources [14, 20]. Natural and anthropogenic causes of wild vegetation loss and transformation of cultures further exacerbate the situation in most parts of Ethiopia including Delanta. Hence, promoting the cultures and the LK are vital for halting the loss, shaping and conserving the floristic diversity. Notably, attempts to respond to healthcare issues lead to ethnobotanical documentation on TMPs [9]. However, as the ethnobotanical information is not documented and remains in the memory of elderly practitioners and end users, the knowledge base continues to be threatened [21, 22]. Adequate information on the TMPs of Ethiopia could only be obtained when studies are undertaken in the various parts of the country where no ethnobotanical explorations have been made [23].

Maintenance of the balance between conservation and human needs has always been a complex matter. Environmental resettlement programs have been positive with respect to alleviating the problems of recurrent food insecurity and enhance restoration of useful wild plants in the original places from where people were moved out. This, however, requires careful evaluation of people's attitudes and perceptions of LK [24, 25]. Resettlement took place in Delanta at different times [17, 18, 26, 27] and the resettlement is still continuing. More floras, however, were lost by drought, further aggravated by returning of thousands of resettlers; recovery efforts have been even more difficult [19]. As a plant species is lost from a locality, the information contained in it will also be slowly blurred and finally become lost forever. Urgent ethnobotanical studies and subsequent conservation measures are, therefore, required to salvage these resources from further loss. Thus, the main objective of this study was to document the availability of plants which have been used as remedy to combat ailments and the LK on use of these resources together with the impacts of the 1984/85 resettlement as so far no such study has been conducted in Delanta.

## 2. Material and Methods

### 2.1. Description of the Study Area and Climate

**Figure 1:**
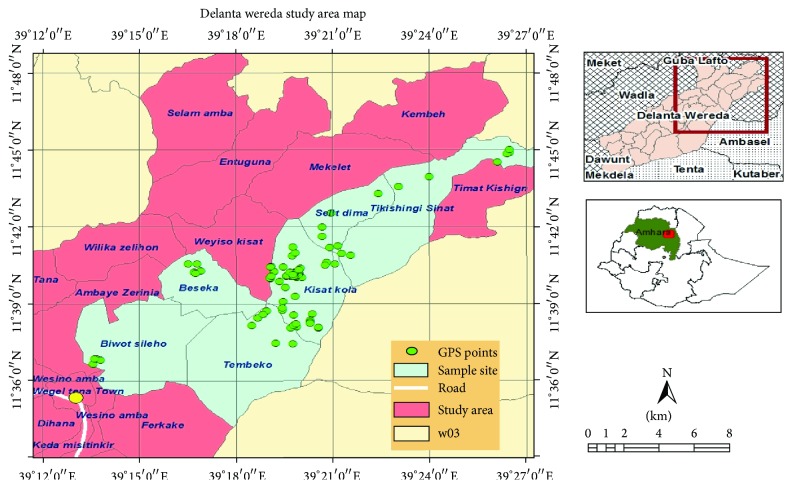
Map of Ethiopia showing the location of Delanta district and the study sites.

Delanta district is located at 530 km north of Addis Ababa in northwestern Wello. The major town, Wegeltena, is situated at 11°35′N latitude and 39°13′E longitude with an elevation of 2555 m (Figure 1). The district has an estimated total population of 128, 416 with an area of 1060.17 square kilometers. The largest ethnic group is the Amhara (99.96%) and Amharic is spoken as a first language [[28], Delanta District Agricultural and Rural Development Office, unpublished annual report of 2011]. The Health Centre of Delanta District has eight health stations and 32 health posts. However, access to modern health services is very limited in both personnel and equipment availability. Therefore, as Abebe et al. [13] indicated, a better alternative for the majority is to use the traditional herbal remedies. The statistical data of the Delanta District Health Centre [unpublished annual report of 2011] shows that, among 20 types of diseases, the most frequently occurring are helminthiasis, lower and upper respiratory tract infections (LRTI and URTI), and arthritis.

**Figure 2:**
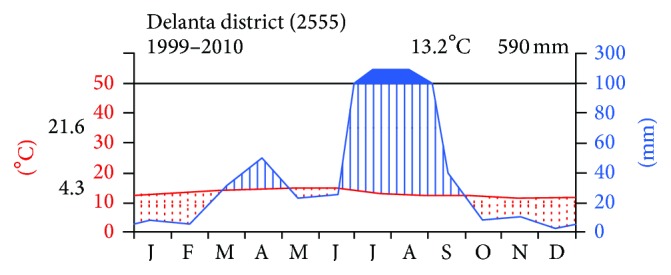
Climadiagram of the study area from the years 1999 to 2010 (data source: National Meteorological Service Agency of Ethiopia, Kombolicha Branch Directorate, 2011).

The study area receives bimodal rainfall. The major peak is important for crop production when the annual rainfall is received from mid-June to the beginning of September. The climadiagram in Figure 2 illustrates that mean annual rainfall and the temperature are 590 mm and 13.2°C, respectively. As the climadiagram and some authors [14, 29] indicate, the natural vegetation of the study area largely falls under the dry ever green montane forest occurring in the altitudinal ranges of 1,500 to 3,200 m. However, the intense deforestation of the highlands led to massive loss of forest cover. One of the main contributing factors to the weakness of environmental protection and its proneness to drought and famine has been the deterioration of the natural vegetation.

The district is divided into four agroecological zones. Farmers depend on both BELG and MEHER rainfall. Although these two production systems vary in source of income along with elevation, crop production is the largest source of income, followed by livestock and off-farm sources in the district [18, 28]. With regard to geology of Delanta, very stable and excellent qualities of Ethiopian opals are found in a specific alternating layer of basalt and rhyolitic ignimbrite thick volcanic series [30]. However, fatal accidents and loss of biodiversity (wild useful plants) caused by collapsing rocks or falls from cliffs have been reported and observed. Thus, the important solution to balance the mining benefits and the loss of biodiversity could be replantation of trees in the mine areas.

### 2.2. Ethnobotanical Methodology

Ethnobotanical information on the traditional use and management of TMPs was collected through participatory rural appraisal involving semistructured interviews and focus group discussions. All the discussions and interviews were conducted in Amharic language. The impact of the 1984/85 resettlement on vegetation rehabilitation was examined with guided field walk. Market survey was integral part of this research. Purposive sampling method was used and six representative sites (*kebeles*) (kebeles are the smallest administrative division next to district) consisting of 38 villages were selected within the district. Most of the sites were in the middle agroecological zones of the eastern part where the 1984/85 famine was especially worse [18, 26]. In total, 72 informants aged 20 to 88 years (55 males and 17 females) were selected, of whom 18 key informants were selected by purposive sampling based on recommendations of elders and local authorities from every six study sites with equal numbers.

Plant specimens were collected with local names and GPS data, pressed, dried, and brought to the National Herbarium (ETH), Addis Ababa University, for final determinations and confirmation using taxonomic keys in the flora of Ethiopia and Eritrea, comparison with authentic specimens, and expert assistance. The voucher specimens with labels were then deposited at the ETH. The vegetation of the study area was described using both the emic and etic categorization methods. The dominant or associated codominant species gave etic plant community types and the emic categories followed Martin's system of emic vegetation classification [1], which relied on the way the people perceived plants and gave names in Amharic language.

The collected data were analyzed using descriptive statistics to evaluate the percentage and frequency of different aspects of TMPs. Preference ranking was conducted by using nine randomly selected key informants to rank five TMPs' use against febrile illness and the degree of scarcity of other five TMP species. In paired comparison, nine key informants were asked to choose the top five TMPs used to treat stomachache based on their medicinal values. The number of pairs for each was calculated by applying the formula: Number  of  pairs = *n*(*n* − 1)/2, where *n* is the number of items. The total number of items was obtained by summing up the total scores obtained. Direct matrix ranking was used to compare seven multipurpose TMPs and six principal threats using nine key informants following Cotton [5]. Informant consensus factors were calculated for each ailment category to identify the agreements of informants on the reported cures using the following relationship: ICF = (Nur − Nt)/(Nur − 1), where Nur is the number of use reports from informants for a particular plant use category and Nt is the number of taxa that are used for plant use category for all informants. Eight categories were identified based on local explanation. ICF value ranges from 0 to 1. Abundance score was conducted to reflect the trends of indicator species in homegardens and wild vegetation during periods from the 1970s to 2011. The informants were asked to score the availability of an indicator species of TMPs. The scores ranged from 0 to 2 where 0 reflected none or nearly none; 1 a few or some; 2 many or readily available following Nanyunja [20].

## 3. Results

### 3.1. Diversity and Growth Habits of TMPs

In total, 133 TMP species (including two vascular seedless plant taxa) distributed into 116 genera and 57 families were documented. Those species were collected from different habitats, notably wild vegetation that could be enclosed and unenclosed, farmlands, and homegardens. The growth form analysis of total TMPs indicated that the most widely used plant remedies are obtained from herbs (71 species, 53.4%), followed by shrubs (48 species, 36.1%) and trees (14 species, 10.5%). Of the total 57 families, Asteraceae and Lamiaceae were found to be represented by the highest number of species (14 and 12, resp.), followed by Euphorbiaceae and Solanaceae with eight species each. Three families had four to five species, another five families had three species each, 16 families had two species each, and the remaining 29 families were represented by one species each (see Appendix  1 in Supplementary Material available online at http://dx.doi.org/10.1155/2016/5060247).

**Figure 3:**
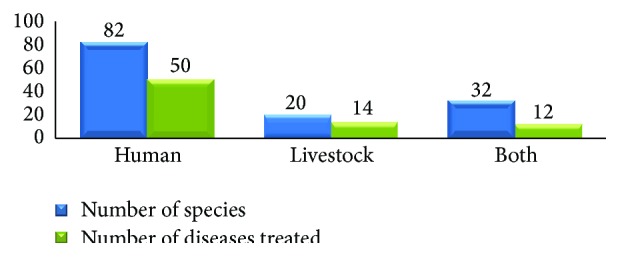
Proportion of treatment of human, livestock, and both human and livestock ailments.

*TMPs Used for Treating Human and Livestock Ailments*. Of the reported 133 TMP species, 82 (61.7%) species representing 72 genera and 44 families were reported as medicine for treating human ailments (Figure 3). Generally, all the TMPs were used for the treatment of about 76 ailments of human and livestock (Table 1).

With regard to plant parts, leaves were the most frequently used parts (32.6%) used to treat various ailments, followed by roots (21.7%), and further details are given in Table 2. Most frequently used preparation methods of TMPs in the area are chopping, crushing, and pounding, which account for the highest proportion (38.3%), followed by grinding in powder form (20.0%); roasting, boiling, and cooking (11.3%); rubbing and unprocessing (11.3%); squeezing (10.8%); and chewing and absorbing (8.3%). Once herbal medicaments were prepared, dosages were estimated using different locally available materials. Doses of liquid remedies were estimated by using plastic cups or glasses (could be coffee-cup, tea-cup, or water-cup) or gourd utensils, lid of rubber, or number of drops. Spoons (teaspoon) for powders, counting numbers for seeds and fruits, and index finger size for roots are some of the traditional tools of estimation.

After estimating the doses, different routes of administration were used. Oral route (43.9%) was being the most common route of administration, followed by dermal route (28.7%) as shown in Table 2.

In Delanta, no side effects were reported by informants except some species like* Calotropis procera*,* Euphorbia* spp., and* Lobelia rhynchopetalum*. The white latex of these species was used for treating different ailments, which was reported as noxious for humans if not properly handled.* Hagenia abyssinica* and* Silene macrosolen* were said to result in vomiting and diarrhea and if the risk is diagnosed, patients were given SHIRO WOT (sauce made of pulse grains) and powder of* Linum usitatissimum* infusion in water to reduce the pain.* Cucumis ficifolius* and* Phytolacca dodecandra* were said to have similar counter indications unless proper care is taken in dosage determination. Milk products, salt, honey, coffee, traditional ale, food, and water are some additives used by healers when preparing remedies to improve the taste and ointments of remedies. Most of the plant remedies were employed in fresh forms (54.4%) followed by dried (26.5%) and both fresh and dried forms (19.1%). The local people stored dried remedies for future use only for some diseases. The majority of prepared remedies were applied through eating, sucking, decanting, and inhaling (internal application, 67%), followed by creaming and tying (external application, 33%). 

*Consensus on Use, Popularity, and Importance of TMPs*. The top ten TMPs were known by more than one-fourth of the informants.* Ocimum lamiifolium* is the most popular, cited by 66 informants (91.7%) for its medicinal value, followed by* Zehneria scabra* with 61 (84.7%), and others are given in Table 3.

Febrile illness was the third common disease of both human and livestock in the district health office. Preference ranking of five TMPs was reported as effective for treating febrile illness.  Table 4 shows that* Ocimum lamiifolium* and* Zehneria scabra* came in the first and second ranks.

Paired comparison was made among five TMPs that were identified by the informants to be used in treating stomachache (Table 5), which was the 2nd common human disease according to data from the district health office. The results showed that* Schinus molle* and* Verbena officinalis* ranked first and second.

*Informant Consensus Factor (ICF)*. Diseases that were found to be prevalent in the area were treated by a variety of TMPs. The highest ICF value (0.91) has been shown from the categories of respiratory disease, febrile illness, and throat infection with relatively smaller number of species (19) used by a large proportion of the healers, followed by disease related to internal parasites and gastrointestinal disorders with ICF value of 0.82, represented by the highest number of both species (46) and informants (248). On the other hand, the category of genitourinary problems was only treated by healers, which had the lowest ICF value of 0.43 with five species (Table 6). 

*Marketable TMPs in Delanta District*. Market survey was conducted at Tirtria and Adagua in the major town. Some herbal medicinal plants were recorded based on direct observation and interviewing traders, vendors, and consumers. They reported that most of the herbal medicines were sold and bought for various use values such as spices, condiments, foods and beverages, fumigants, and cultural and spiritual aspects. These species included* Allium cepa*,* A. sativum*,* Capsicum annuum*,* Myrtus communis*,* Rhamnus prinoides*,* Cucurbita pepo*,* Olea europaea* ssp.* cuspidata*,* Otostegia integrifolia*,* Ruta chalepensis*,* Vicia faba*, and* Zingiber officinale*. 

**Figure 4:**
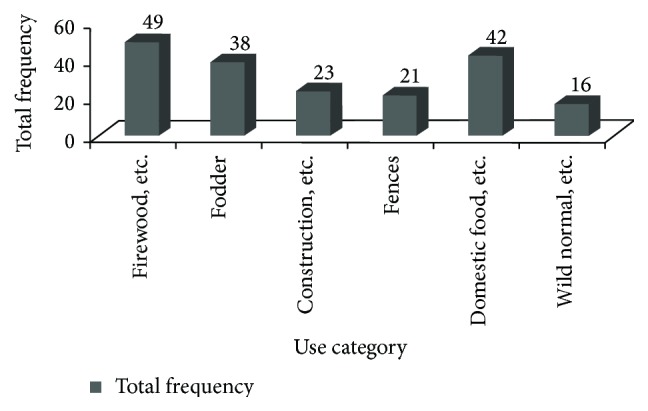
Proportion of other uses of the ethnomedicinal plant species.

*Multiple Uses and Ranking of Ethnomedicinal Plant Species*. Some of the TMPs in the study area were used for other purposes. Multiple use analysis showed that firewood and charcoal are the most frequently mentioned uses (27.3%) while the wild (normal and famine) food category was the least reported (10.0%) (Figure 4).

Use diversity analysis shows that, among the six TMPs,* Carissa spinarum* is ranked 1st, followed by* Rhus vulgaris* ssp.* neoglutinosa*, and the others are given in Table 7.

*Threats to and Conservation Status of TMPs*. The threats resulted mainly from human activities and they varied from site to site. Among these activities, agricultural land expansion is ranked 1st, followed by fuel wood and construction material collection, overgrazing, mining opal, and drought (Table 8).

Further analysis showed that* Withania somnifera* came out in the first rank of most threatened TMP (Table 9). 

**Figure 5:**
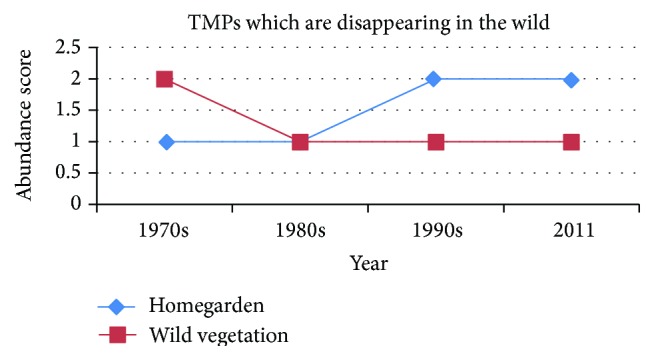
Trends of ethnomedicinal plant species in homegarden and wild vegetation.

*Trends in Abundance of TMP Species*. The degree of abundance of TMP species considering their current status was reported as 50 common species, followed by rare (45 species) and sparsely distributed species (40), based on informants' perception and direct field observation in the wild state.  Figure 5 shows that the homegarden is maintained in an increasing state of some of the indicator TMP species, particularly those said to have been lost from the wild environment in the 1970s, and then went on decreasing. 

*Local Ecological Knowledge of People*. In the study area, people have knowledge about their environment (landscapes, vegetation, and soil types). Based on topography of the land forms and the color and fertility of soils, they classified the ecological units into six categories in Amharic language based on their lifelong LK. This is presented along with the etic categories in Table 10. 

*The LK of People on Plant Community Type and Distribution of TMPs*. Although natural forests have disappeared in many places due to many reasons, the vegetation of the study area still keeps relatively high number of species of TMPs. However, among 19, six endemic plant taxa in the floristic region of Ethiopia and Eritrea were not recorded as they are found in the floristic region of Wello (WU). These are* Urtica simensis*,* Impatiens rothii*,* Aloe pulcherrima*,* Solanum marginatum*,* Lobelia rhynchopetalum*, and* Primula verticillata* ssp.* simensis*. The plant community types were identified through categorization of the local people (emic classification approach) and observation of the researcher (using etic classification) based on the dominant plant species as visually inspected. Eight major community types were recognized. All types of plant community types are found in six study sites except* Lobelia rhynchopetalum* and* Euryops pinifolius* dominated community type, which is restricted in one of the study sites, namely, Tikshign Sinat kebele between 3539 to 3702 m a.s.l. Some of the following species are not included in the present paper, but they are collected and preserved in ETH by the authors for other purposes (Table 11).

**Figure 6:**
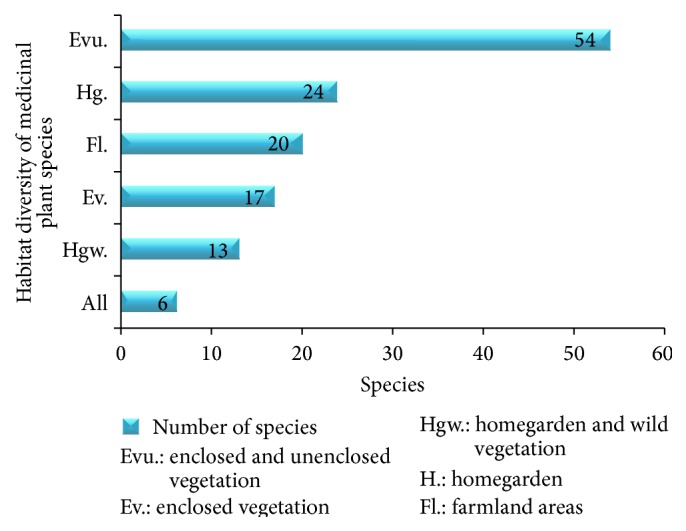
Proportion of habitat diversity of TMPs.

The present study noted that well-organized emic classification of local vegetation was not shown in some ecological settings (largely DEGA zones) where natural vegetation has been almost completely changed into agricultural lands. Regarding habitat, most of the TMPs are distributed in different habitats though their availability varies from place to place among species. The majority were harvested from the wild (71 species), followed by homegarden (24 species), and cultivated in farmlands (Figure 6). The wild vegetation could be either enclosed (protected by government or private sectors) or unenclosed, which is open to all people.

### 3.2. Comparisons of LK among Age Groups and Its Transfer System

**Figure 7:**
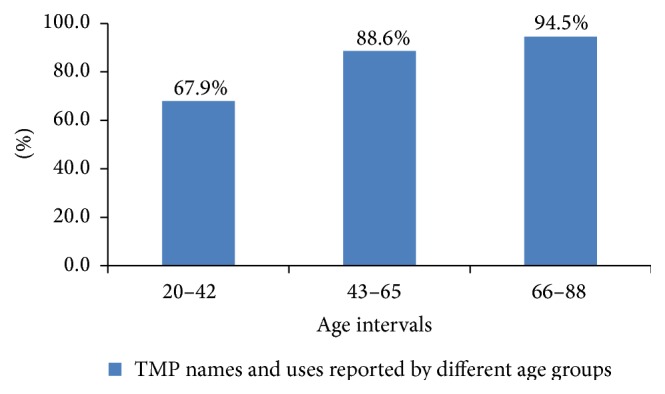
Variations of LK in TMPs among the three age groups of informants.

The LK of the three age groups was compared with respect to the names and the respective uses of TMPs. The sample sizes of each age group were 14, 28, and 30 persons from 20 to 88 with 22 years' gap. The results show that the age groups within the ranges of 66–88 years reported the highest proportion of TMP names and uses. The total value is more than 100% because sometimes same plant species were mentioned by all groups (Figure 7).

The majority of traditional healers (84.4%) kept the knowledge with them and selected family members for the sake of confidentiality while others (16.6%) transferred their LK to other persons (Table 12).

*Consequences of Resettlement on Environmental Component and LK of TMPs*. The study reported that half of the interviewed informants were those that returned from the destination area due to the 1984/85 resettlement. This indicated that the 1984/85 drought was one of the worst disasters in the area. In case of returnees, 17 plant species have no names and some of the names given also were more general (e.g., HAREG refers to any climber plant). However, this is not seen in traditional healers who have used “medicoreligious manuscripts.” In case of resident people, however, some of the TMPs are named by using the disease treated adding YE at the beginning followed by MEDANIT. For example, three species (*Cistanche tubulosa*,* Polygala abyssinica*, and* Vernonia schimperi*) are named as YESATMEDANIT, YEBABMEDANIT, and YEMICHMEDANIT, to say medicine of burns, snake bite, and febrile, respectively. It is noted in Table 13 that permanent inhabitants have complete names for each except two species (*Ekebergia capensis* and* Pulicaria schimperi*), which is not the case for the remaining groups of informants.

**Figure 8:**
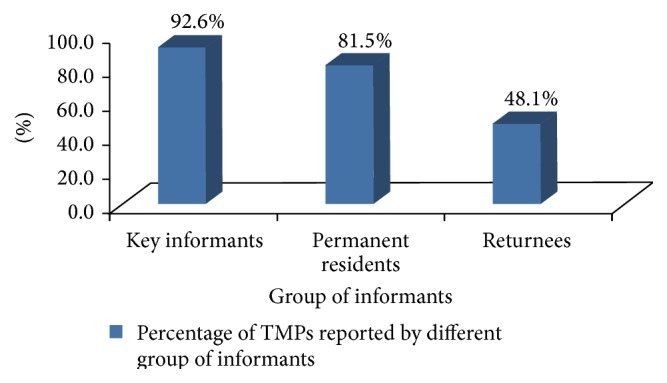
Variations of LK of TMPs among three groups of informants.

Of 36 returnees including some key informants, only two persons were born in the destination of resettlement areas and the rest were primarily born in the original area and then left from their original area during the 1984/85 famine. However, after staying away at different times, they came back to their original location (Delanta district). The sample sizes of the three groups of informants were 18 key informants, 25 permanent residents, and 29 returnees. The results showed that key informants are more knowledgeable about TMP species (92.6%), followed by permanent residents (81.5%). However, concerning returnees, the least results were recorded in all the three aspects (Figure 8).

The findings on the impacts of resettlement depict that the 1984/85 resettlement had both positive and negative results in the area of ethnobotanical knowledge and social and cultural activities. From negative perspective, for instance, majority of the returnees had lost specified local names and detailed preparation of TMPs, and youngsters were not willing to respect the LK and the associated taboos. For the positive perspectives, on the other hand, the former farmlands were converted into noncultivated vegetation. Such promoting ecological rehabilitation in turn provided restoration of wild useful plants and reduced human pressure on plant resources.

## 4. Discussion

Relatively high number of TMP species is encouraging and a good indication that the area has reasonable number of useful plant species. This is partly the result of the action to move out drought-affected people from the area and the fact that the permanent residents went on using and protecting the plants. The results agree very well with the findings in the Cheffa plain of southern Wello where 83 TMPs species were recorded [31]. Although there is continued deforestation and degradation in the area, the remaining species and the taxonomic diversity (59 families) promise success in conservation. As in other studies made in southwestern Ethiopia [22] and elsewhere [8], the family Asteraceae came up with high number of species. Asteraceae is also the second (next to Fabaceae) largest represented dicotyledonous family in the flora of Ethiopia and Eritrea with 440 spp. The results of this study prove that people tend to use preferably the plants that are easily available to them excluding, of course, those that are toxic or lethal. As was affirmed by other studies [8], the more common the plant species is in an area, the greater the probability of its popular use is. The study showed that the wild vegetation is the major habitat where a significant proportion of TMPs are harvested. The distributions of all, but two (IV and V), of the plant community types also confirmed this habitat. This result is in agreement with different studies carried out elsewhere in Ethiopia [7, 23] and other parts of the world [11].

The people of Delanta rely largely on herbs, which are replacing the forest resources and are relatively common in the areas where extensive degradation has taken place. This finding agrees with findings of many authors [12, 21, 32] but disagrees with other studies [31, 33]. This could be related to the level of agricultural expansion and perhaps due to the presence of two rain-fed seasons. In most cases, there are several plant species used in combination to treat specific ailments, which was considered important to increase the strength and effectiveness of the remedies. Such practice was also common in other countries [34]. According to Abebe and Ayehu [6], the use of several species for the treatment of a particular ailment could indicate the prevalence of the species. The common use of leaf for preparation of remedies could partly be due to the relative ease of finding and simplicity of preparation. Leaves were shown to be the most commonly utilized parts in other findings [12, 22, 31]. Contrary to this finding, Hunde et al. [33] reported that roots were the most widely used parts, and this may be related to the medical culture of the people and environmental condition of the area. Moreover, collecting leaf parts for medicinal purpose is usually not a threat to the survival of plants as compared to the use of whole parts, roots, and stem barks [6, 35].

Among several preparation methods, the most frequent use of chopping, crushing, and pounding could be because of ease of use of local tools. Similar finding was reported by Tamene et al. [31] but deviates from other findings [12]. Informants underlined that attention is given in determination of dosage and depends on experience of traditional healers, age of patients, and the power of TMPs. They also had relatively better measurements for humans than livestock. However, lack of precision and standardization in measurement is considered as the general weakness of traditional healthcare system [4, 32]. The findings showed that most remedies in Delanta were administered orally in agreement with other reports from northern Ethiopia [6, 31, 32]. The fact is that modern health system also reported that there was high prevalence of internal problems and gastrointestine disorder in the study area. This also emerged from the calculations of ICF values. However, Giday et al. [22] indicated that skin (dermal) was the predominant route of administration. Most remedies were prepared and used immediately after harvest. As traditional healers pointed out, the use of either fresh or dry form is based on the type of applications and the availability of TMPs. Herbal drugs were mostly applied internally (58.3%) and the plants were found around homegardens. Fresh forms were favored since this form is considered to be strong and healthy. The wider use of fresh conditions was also reported from elsewhere [22, 35].

In the community, some of the TMP species were more popular and recognized as more effective and popular remedies than others. From both rankings and comparisons, it could be understood that the most favored species are usually the most effective ones for being used against a particular ailment. Thus, the preferences of some TMPs more than others prove the reliability and continuity of the ethnomedicinal information obtained from the local people. Among eight identified disease categories in the area, respiratory diseases, febrile illness and throat problems, and internal parasites and gastrointestine disorders were the most frequently encountered with high value of ICF. This may indicate high incidence of these types of disease in the area, possibly due to the poor socioeconomic and sanitary conditions of the people perhaps related to drinking stream water and food preparation. This finding is in line with another study conducted in northwestern Ethiopia [32]. Heinrich [36] also indicated that TMPs that are believed to be effective in treating a certain disease have higher ICF values whereas a low value implies that the informants disagree on the taxa. All these signified that people rely chiefly on TMPs to meet their primary healthcare needs and this holds true in other previous studies [6, 12]. Some of the species discussed herein (Table 1) were also incorporated in modern pharmacological remedies with the same treatment and parts used by other researchers [37–39]. These are good indicators that the present study will serve as an input for further works in modern therapeutics activity.

With this study, informants noted that resettlement program contributed to biodiversity conservation in the original place as evidence of the TMPs that are regenerating in the wild vegetation and former farmlands since the local people left Delanta. This was mainly seen in the lowlands (1700–2600 m a.s.l.) of the study area that was hard hit by the 1984/85 drought as described by Rahmato [26]. Inappropriateness for farming is also served as reinforcement for the areas that have been shifted to restoration and unsettled lands. Other findings were reported by different authors elsewhere in Ethiopia [25, 40, 41] also affirmed that the basic rationale behind resettlement was to facilitate resource rehabilitation and to provide poor households with a better livelihood. According to Dhakal et al. [24], however, it needs careful evaluation of peoples' perceptions on volunteerism to handle with proper management of biodiversity.

Although many positive outcomes were seen in the recovery of habitats, there was some loss of LK and culture of resettled people after resettlement. Such mixed results were also shown in another study [24]. Informants reported that before the 1984/85 resettlement people's judgments on their conservation outcomes of vegetation which foster TMPs were very positive. With regard to returnees, however, this habit has been reducing so that resettlement (staying away from the original site) could be one of the reasons. Traditional healers who might not have gotten opportunities to come back to their original place may have lost some of the LK to treat ailments such as asthma and tuberculosis (lung problem) in the local people was mentioned. This was again another barrier in transfer of knowledge to the young generation. This resulted in the loss of specified local names of some plants and their uses. According to some key informants, some TMP species were lost by improper exploitation of root parts which was aggravated by drought: HAKENUR, WEYILO, SIREBIZU, SHEBOTETYA, TIFRENA, and CHOCHO (in Amharic). Similarly, another study in the same area [19] pointed out that recovery efforts of the lost floras have been even more difficult. Some of the returnees are encroaching and clearing both rehabilitated and natural vegetation which harbors TMPs unlawfully. Another influence of resettlement was that economic crisis of returnees—those who were totally dependent on safety net program.

Out of total 133 collected species, 17 (13%) species are endemic TMPs in the flora of Ethiopia and Eritrea. As it holds true for the total species, Asteraceae is also the leading family with five species, followed by Lamiaceae with three endemic species. These were cross-checked with different volumes of flora of Ethiopia and Eritrea and Red List book [42]. Eight of these endemic and a few other nonendemic TMP species are not described as they have been distributed in Wello floristic region (WU) in the flora. IBC [14] and Friis et al. [29] mentioned that the dry evergreen montane and grassland complex ecosystems cover the major part of the study area. Additionally, the present study revealed that the Afro-Alpine and sub-Afro-Alpine ecosystems are also found in some mountain areas and characterized by the most conspicuous endemic giant herb (*Lobelia rhynchopetalum*) and shrubs including* Euryops pinifolius*, the evergreen tree heather (*Erica arborea*), and perennial herb species in the study area. Hence, there is a need to strengthen conservation actions in these ecosystems to stop further threats of endemic species.

Based on the reports of informants, the trends of indicator species revealed that the last 40 decades were much detrimental to natural vegetation in the area. Furthermore, wild plants used in TMPs are being lost more quickly in wild vegetation than homegardens as illustrated in Figure 5. All the indicator TMP species which are threatened by different factors (Table 8) were reported to be greatly harvested for multiple uses they provide. The root parts used as medicine could be posing another threat. There are 113 multipurpose plants which have diverse services in terms of economical, ecological, and cultural aspects other than their medicinal values (Figure 4). The results of data matrix ranking of TMPs on five main uses showed that all the six species were the most favored for firewood although they are overexploited for other multiple uses. This goes in line with other findings [5, 6, 10, 11]. Thus, conservation strategy is needed in the area to save these species from further reduction or total extinction due to unsustainable use and overexploitation.

In view of long human settlement history of the area, the natural vegetation has enormously been altered. It came out clearly from the study that the threats facing TMPs are both anthropogenic (e.g., agricultural expansion, which is ranked 1st) and natural factors (e.g., drought), which are having detrimental effects on wild medicinal resources. This finding is in agreement with other studies conducted elsewhere in Ethiopia [7, 43]. The previous studies conducted elsewhere also confirmed that LK of wild plants in Ethiopia is in danger of being lost, as habits, value systems, and the natural environment change [6, 7].

When the pressures of threats increased and some multiuse plants became rare in the wild vegetation, most traditional healers and some local people started to conserve them by growing such species intentionally in their homegardens and farmland margins. This report is consistent with that of Asfaw and Tadesse [7], as they explained that homegarden is a strategic farming system for conservation and enhancement of TMPs. The other ways by which conservation of useful plants is effected relate to the culture of people themselves and some taboos. For instance,* Phytolacca dodecandra* and some* Solanum* spp. were not used for firewood, because the local people believe that those who used these species for firewood become poor and divorced from spouses. Furthermore, cutting of big trees with large umbrella branches was culturally not accepted because of the belief that one who cuts them will have a shortened life and may soon pass away. This is widely seen in plants like* Ficus* spp. (YETINCHAW LEMLEM),* Euphorbia abyssinica*, and* Hagenia abyssinica* (ADBAR-ZAF) related to spiritual issue. It also holds true for sacred forests which are found around monastery and church yards. Such habit of conserving useful plants is still alive in the study sites but is ageing in the majority of present generations. Likewise, Tamene et al. [31] reported about the preservation of forest islands by the community's sociocultural factors as rituals. Therefore, incorporation of local communities' own vision and LK concerning conservation and sustainable use of TMPs has to be given priority. It is likely that the local people closely watch and know how the resources are consumed and conserved [20, 44].

It is obvious that overgrazing and deforestation are very serious in northern highlands of Ethiopia. Presently, however, the local people have practiced check dam construction and tree plantation with enclosed vegetation of such highlands to reduce erosive rain storms and to preserve useful plants as well. The significance of TMPs to people can be sufficiently great that arrangements made for the conservation and sustainable use of TMPs have now grown to be a timely issue in Ethiopia. In general, biodiversity has to be protected for its multidirectional values [45, 46].

Though the local people are exposed to high cultural and habit change due to the repeated drought and resettlement conditions, the ethnobotanical knowledge on classification of their ecology and diverse use of TMPs were transmitted orally through generations. Different studies affirmed that the local people are knowledgeable about their environment in general and plants in particular [1, 33]. The findings of Giday et al. [22] indicated that boys were usually favored. Likewise, 50% of free transfers of knowledge particularly took place from parents to sons in this study. Similarly, several researchers reported that the distribution of LK is hierarchically placed and the services are obtained from the family, the village, or beyond. The secrecy of LK on medicinal practices could be one of the reasons for the uneven distribution of LK of TMPs in Ethiopia among community members [7, 21].

Comparison of LK on TMPs among age groups proves that knowledge on TMPs is wider among elderly persons while the youngsters are comparatively less knowledgeable. Since majority of young age groups are educated, modern education might have made the young generation underestimate the traditional practice. This is in line with the study of TMP in Kafficho and Butajira people [23, 47], which showed that illiterates and older residents are more likely to use traditional medicine than the educated and youngsters. Thus, age and education are main factors that appeared to influence the use of LK. On the other hand, the LK on the use of three groups of plants also varies among three groups of inhabitants. In fact, the knowledge of key informants on utilization of TMPs is proportionally high (92.6%). It is noted that the knowledge of TMPs is a means of income generation for the healers in the study sites. The folk naming of the plants by permanent residents, usually associated with their function and traits, is consistent with another study conducted by Awas and Demissew [23], which indicated that some names are attached to the disease treated and some are attributed to domestic or wild animals. However, returnees are the least knowledgeable and the names they gave to the plants were not specified. This could be due to the absence of returnees at the time of famine (1984/85). Furthermore, Martin [1] described that the knowledge on plant uses changes with time and space and with change of resources and culture. Therefore, the expansion of modern education and resettlement of people (to elsewhere) have resulted in the deterioration of LK and practices with the dislocated members of the community.

## 5. Conclusions

The present study indicated that the local people of Delanta are custodians of large number of TMP species (133) that they named and explained for the treatment of various human and livestock ailments (76). Notably, the highest proportion of TMPs (65.8%) were cited for human ailments. Various ethnobotanical analytical tools showed that the local people preferred some species over the others in treating ailments and other uses. About 85% of these resources also provide multiple uses. While most of the TMPs are harvested from the wild vegetation, the area is exposed to many threats such as agricultural expansion and other human induced pressures. As a result of these factors, some of the plants and the associated LK are under threat and declining. The ethnobotanical knowledge on TMP species varied among key informants, permanent residents, and returnees. Returnees were the least knowledgeable and this is one of the negative consequences of resettlement. The findings in general indicate that resettlement provided an opportunity to improve the restoration of useful plants and proved to be advantageous to biodiversity conservation in the original location (Delanta) and people should be resettled within their close community in order to avoid any disruption of their LK.

Homegardens and farmland margins have contributed to serving as preserving places of species presently in short supply and this is in need of further enhancing. Thus, strengthening the conservation of TMPs in such places is very important. For better diversity of species in general and TMPs in particular, in situ conservation measures need to be particularly directed to plant community types which could allow conservation of 50% of TMPs (*Becium grandiflorum* and* Rumex nervosus*;* Otostegia integrifolia* and* Dodonaea angustifolia* dominated community types). These potential TMP species may even give an insight for further pharmacological and therapeutic development in Ethiopia.

## Supplementary Material

A total of 133TMP species belonged to 116 genera and 57 families were documented in Delanta. Those species were collected from different habitats where they are varied in altitudinal ranges from 1802 to 3702 m a.s.l., growth habits and species abundance distribution. The botanical names of all species with authorities and their local names are given in Appendix 1.

## Figures and Tables

**Table 1:** TMPs used against human and livestock ailments and detailed information on methods of preparation. Description of data: Uf.: used for (L: livestock; H: human; Hl.: both), Cp.: condition of preparation (F: fresh; D: dried; and Fd.: fresh & dried), Ra.: route of administration (D: dermal, Op.: optical, N: nasal, O: oral, A: anal, and V: vaginal), and Pu.: plant parts used (R: root, S: stem, Sb.: stem bark, Rh.: rhizome, L: leaf, F: fruit, Fl.: flower, Se.: seed, B: bulbs, La.: latex, W: whole parts, and Ag.: abovegroufnd part).

Scientific name	Pu.	Uf.	Disease treated	Ra.	Cp.	Method of preparation and application of TMPs
*Achyranthes aspera*	L	H	Conjunctivitis	Op.	F	Pounded and squeezed leaf juice is filtered with cotton cloth and dropped into the eye
	L	H	Impetigo (*Kunchir*)	D	F	Crushed leaf is bandaged on the wound
	R	H	Hemorrhage at birth	O	F	Pounded and squeezed leaf is filtered with water to be drunk in a half size of water cup
	R	Hl.	Bleeding of any part	D	F	It is crushed and then dressed on the bleeding part of livestock and human

*Aeonium leucoblepharum*	L	H	Emergency (trauma)	D	F	Crushed leaf juice is creamed on head and face or any parts that felt sickness

*Ajuga integrifolia*	L	H	Tonsillitis	O	F	Pounded and squeezed leaf juice is drunk with a coffee glass

*Allium cepa*	B	L	Leech infestation	N	Fd.	It is crushed and mixed with water and poured into the nose in both openings
	B	H	Leech infestation	O	Fd.	It is crushed and mixed with water and then poured orally

*Allium sativum*	B	H	Influenza virus	N	F	The bulb is peeled and the aroma sniffed
	B	H	Dry cough	O	Fd.	The bulb is chopped and mixed with ground seed of *Guizotia abyssinica *and* Nigella sativa *powder form of* Thymus schimperi *and crushed* Zingiber officinale *is drunk continuously
	B	H	Evil eye	D/N	F	Its bulb with the roots of *Withania somnifera, Lobelia giberroa, Sida schimperiana, Carissa spinarum, Dodonaea angustifolia, Verbena officinalis, Capparis tomentosa, Croton macrostachyus, Verbasicum siniaticum, Jasminum grandiflorum, Ceratostigma abyssinicum, Clerodendrum myricoides, Ferula communis, Cyphostemma adenocaule*, and* Cucumis ficifolius *and whole parts of* Artemisia afra, Ruta chalepensis*, and *Lepidium sativum *are crushed and the smoke on the burning firewood is sniffed; the powder form is tied on the neck
	B	H	Heartburn (*Tkusat*)	O	F	Chopped bulb is boiled in water and then the decoction is drunk in a tea glass

*Aloe camperi*	Fl.	H	Idiopathy (*likift*)	D	F	It is cooked with chopped root of *Impatiens rothii *and flower of* Buddleja polystachya *and then the irritated feet and fingers are immersed in the cooker for three consecutive days
	L		Evil spirit at birth	N	D	It is placed on burning dung with *Otostegia integrifolia* and fumigated the house and is inhaled

*Aloe pulcherrima*	La.	Hl.	Wound (irritation)	D	F	It is pasted the latex on the wounded part of livestock and human
	R	L	Rh factor/disease	O	D	It is pounded with the root of *Eleusine floccifolia*, *Asparagus africanus, Verbscum sinaiticum, Trajia cinerea, Ferula communis*, bark of *Myrica salicifolia, *and seed of* Sesamum orientale *with pestle and mortar and eaten with Injera for three consecutive days at 9th pregnancy month
	R	H	Rh factor/disease	O	D	It is powdered with *Asparagus africanus *and *Sesamum orientale *and then two teaspoons are taken with the same size infusion of honey for three days at ninth month of pregnancy period

*Artemisia abyssinica*	L	H	Common cold	N	F	The aroma of leaf is inhaled by inserting half part of it into nose
	S	H	Trachoma	Op.	F	The infected outer part of the eye is heated with roasted portion of stem
	W	H	Evil eye	O/D	Fd.	It is kept in pocket as tooth brush; the powder is tied with others like *A. sativum*

*Arundo donax*	Rh.	H	Birth control	O	D	Chopped and pounded rhizome is mixed with root of *Verbena officinalis*, powder form is homogenized in water, and one coffee glass is drunk once in the morning before breakfast

*Asparagus africanus*	Ag.	Hl.	Swelling (*Agjil*)	D	F	It is crushed and homogenized in water for washing the swelling by saying “Betin”
	R	Hl.	Rh factor/disease	O	D	It is pounded with the root of other species like *A*. *pulcherrima*
	Ag.	H	Circumcision	D	F	The young aboveground part is crushed and the injury is applied as cream with yolk of egg

*Bersama abyssinica*	L	H	Ascariasis	O	F	It is boiled in water with seed of *Vicia faba* for drinking the decoction; the cooked bean is eaten

*Brassica nigra*	Se.	L	Bloating	O	D	It is ground and homogenized in residue of traditional ale with *Lepidium sativum* and decanted
	Se.	H	Abortion of unborn child (*Atnt Kirit*)	O	D	It is ground and homogenized in a cup of water and then drunk by pregnant woman

*Buddleja polystachya*	F	H	Idiopathy	D	F	Its flowers are cooked with crushed root of *Impatiens rothii*, same as *A*. *camperi*

*Caesalpinia decapetala*	L	H	Tinea favus (*Lashign*)	D	D	Crushed and powdered leaf is mixed with butter and then the infected head part is creamed

*Calotropis procera*	La.	H	Hemorrhoids/tumor (swollen anal veins)	A	F	Its latex is used alone or mixed with *Euphorbia polycantha, Ficus palmata, Aloe pulcherrima, Lobelia giberroa*,and squeezed leaf of *Clematis semensis *and is creamed
	La.	H	Piercing by sharper	D	F	The latex is applied as cream on part pierced by sharpen material with the help of needle
	L	H	Boils (furuncle)	D	D	Crushed and powdered leaf is applied as cream with latex of *A*. *pulcherrima* on inflamed part

*Calpurnia aurea*	L	L	Body lice (*kijamr*)	D	F	Its leaf is crushed and stirred in water to wash cattle or calves until removal of lice
	Se.	H	Giardiasis and amoebiasis	O	D	The ground seed is mixed with honey and eaten with three-liter rubber lid size in the morning until recovery, before taking other diets

*Capparis tomentosa*	R/Sb.	H	Evil spirit (sickness)	N	D	The root or stem bark is chopped and placed on the fire and the smoke is inhaled
	R/Sb.	H	Epidemic	N	D	The same methods are used to treat evil spirit and hang the remainder on the roof

*Capsicum annuum*	F	H	Appetite loss	O	F	The pod is chopped and mixed with freshly *A*.* sativum *bulb in small amount of water in the bowls eaten with Injera
	F	H	Malaria (revival)	O	F	The same method is used to treat loss of appetite, prior to taking other diets in the morning

*Carduus schimperi*	R	H	Febrile illness	O/D	Fd.	It is pounded and squeezed by adding water and is taken by a cup of tea and the remnant is creamed
	R	L	Febrile illness	O/D	Fd.	It is pounded by adding water and is decanted by a cup of water and the remnant is pasted
	R	L	Dysentery (*Mentie*)	D	D	The room of sheep or goats is fumigated and the remnants are placed there against “Mentie”

*Carissa spinarum*	L	Hl.	Snake bite	O	F	The liquid is chewed and absorbed soon and for animals its crushed leaf is decanted
	L	H	Rh factor/disease	O	F	It is crushed and soaked in water for one day and drunk in one cup of coffee prior to 9th month of pregnancy period
	R	H	Evil eye	D/N	Fd.	It is the same as used in *A*.* sativum*

*Carthamus lanatus*	L	H	Sexual impotency in men	O	Fd.	It is crushed and pounded with the whole parts of *Trajia cinerea *and root of *Hibiscus eriospermus,* then stirred in local beer, and drunk in one cup of coffee until recovery

*Catha edulis*	L	H	Diuretic	O	F	It is boiled in the pot with water at night and the decoction is drunk in the morning

*Ceratostigma abyssinicum*	R	H	Evil eye	D/N	D	It is the same as used in *A*.* sativum*; the powder is tied in the neck with Abesha cloth and the remnant is inhaled through nose

*Chenopodium schraderianum*	Ag.	L	Coccoides (chicken lice)	D/N	D	Aboveground parts are chopped and the room of chickens is fumigated and the chickens themselves sniffed smokes nasally

*Cistanche tubulosa*	W	H	Fire accident	D	D	The whole parts are crushed and powdered and then the burnt part is creamed with butter

*Citrus aurantifolia* (Christm.) Swingle	F	H	Dandruff	D	F	The acetic fruit is pierced and squeezed and the head is creamed with yolk of egg
	F	H	Ringworm (*Agogot*)	D	F	It is pierced and squeezed; then the affected parts are creamed alone specially faces

*Clematis simensis*	L	H	Impetigo (*Kunchir*)	D	F	The leaf is crushed and squeezed and the affected part is creamed with powder of *Triticum* spp.

*Clerodendrum myricoides*	R	H	Evil eye	D/N	D	It is the same as used in *A*.* sativum*; the powder is tied in the neck with Abesha cloth and the remnant is inhaled through nose

*Clutia abyssinica*	L	L	Leech infestation	N	F	The crushed leaf is soaked in water and decanted into cattle through their nose

*Colutea abyssinica*	S	H	Toothache	D	F	Affected tooth is heated by chopped and roasted stem without contact with other parts
	R	H	Emergency (*Berar*)	O	Fd.	It is crushed and pounded with the root of *Inula confertiflora* and the concoction is drunk with coffee; the roots are dug by hoes with stick of *Olea europaea* ssp. *cuspidata* on Friday

*Combretum molle*	L	H	Malaria	O	F	Fresh leaf is boiled in water and the decoction is drunk in a cup of tea

*Conyza hypoleuca*	R	L	Emaciation	O	Fd.	Its root is crushed together with root of *Inula confertiflora, Echnops hispidus*, and the leaf of *Osyris quadripartita*, * Solanecio gigas*, and whole *Leucas martinicensis* in new *Lagenaria siceraria *as a container with water for two to three days and the concoction is decanted

*Coriandrum sativum*	Se.	H	Stomachache (*Alsha*)	O	D	The raw seed is eaten with small amount of ground salt by the patient

*Croton macrostachyus*	Sb.	H	Ascariasis	O	F	It is chopped and boiled in water with seed of *Vicia faba* and the decoction is drunk and the boiled beans are eaten
	R	H	Evil spirit (Sickness)	N	D	It is chopped and placed on the fire with *C. tomentosa *and *Withania somnifera* and inhaled
	R	H	Evil eye	D	D	It is the same as used in *A. sativum*; crushed and powdered root is tied on the neck
	L	L	Poison insects on Sorghum (*Akara*)	O	F	It is crushed alone or with root of* Cyphostemma adenocaula *and* String hermonthica *is soaked in ale and decanted when cattle ate the leaf of *Sorghum* that contained poisoned insects

*Cucumis ficifolia*	R	H	Removal of retained placenta (*Sing*)	O	F	Freshly washed root is chewed and absorbed by woman
	L	Hl.	Eye injury (inserted materials or hit)	Op.	F	It is crushed and squeezed with the leaf of *Jasminum grandiflorum *by adding small amount of water and dropped to the affected eye and then applied as cream butter for 3 days at night and washed with water in the morning prior to exposure to sunlight
	R	H	Birth control	O	F	It is chewed and absorbed with ripe fruit of *Solanum anguivi *before sexual intercourse at the end of menstrual cycle
	R	H	Gonorrhea (*Chebt*)	O	D	In powder form, one teaspoon is infused into doro wot and then eaten by Injera
	R	Hl.	Rabies virus	O	D	Powdered root is mixed with cheese in one cup of tea and then drunk
	R	H	Jaundice (*Yewuf*)	O	D	Powdered root is mixed with cheese in one tea glass and then drunk

*Cucurbita pepo*	F	H	Gastritis (*Cheguara*)	O	F	Peeled fruit is boiled at night and eaten in the morning before taking other diets
	L	H	Dandruff (*Forefor*)	D	F	The head is rubbed and creamed alone or with the leaf of *Datura stramonium*
	F	H	Stomachache (*kurtet*)	O	F	Peeled and boiled fruit is eaten before taking other diets soon by mother at birth

*Cyathula uncinulata*	L	L	Leech infestation	N	F	Crushed leaf is mixed with small amount of water is poured to the cattle via their nose
	R	H	Tapeworm (*Kosso*)	O	Fd.	Crushed and pounded root is eaten with ale/beer of *Hordeum vulgare* before filtration with water, one cup of coffee, for three consecutive days

*Cynoglossum coeruleum*	W	H	Febrile illness (*Mich*)	D	F	The whole parts are crushed and infused in small amount of water and then the parts that felt illness are washed except chest

*Cyphostemma adenocaula*	R	L	Poison insects on *Sorghum* leaf	O	F	It is the same method of preparation and ingredients as used in *C. macrostachyus*

*Datura stramonium*	Se.	H	Toothache	D	D	Seeds mixed to butter are roasted on plate and inhaled the vapor air through opening their mouth
	L	H	Ringworm, dandruff	D	F	The fresh leaf is rubbed and applied as cream on the head like *C. pepo *

*Debregeasia saeneb*	L	H	Febrile illness	O/N	F	It is boiled in water and its decoction is drunk and then the vapor air is inhaled

*Dodonaea angustifolia*	S & L	Hl.	Bone fracture	D	Fd.	Powdered leaf is glued with its cracked stem on the broken bone with sap of *A. pulcherrima*
	R	H	Evil eye	D/N	D	It is crushed and powdered and tied on the neck and roots are inhaled with others same as *A. sativum*

*Dyschoriste radicans*	W	H	Scabies, itching, and injury of skin	D	Fd.	The whole parts are roasted with *Kalanchoe petitiana* and powdered and then the inflamed parts are creamed with butter until recovery but for itching with the leaf of *Rhus retinorrhoea*
	W	H	Wound (*Sheft*)	D	Fd.	It is crushed and pounded with leaf of *Kalanchoe petitiana* and pasted on the injured parts

*Echinops hispidus*	R & S	H	Febrile illness	O/D	Fd.	It is pounded and squeezed and soaked in water and is drunk by a cup of tea and all parts are creamed
	R & S	L	Sunstroke (*Shiwuta*)	O/D	Fd.	It is pounded and soaked in water by a cup of water and is decanted and the livestock is creamed
	R & S	L	Struck cattle	O/N	Fd.	It is pounded and infused in one cup of water and decanted, and fumigated the smoke for cattle
	R & S	H	Epidemic; evil eye	N	D	It is crushed and placed on the hot fire and inhaled by all householders

*Ehretia cymosa*	L	L	Leech infestation	N	F	Crushed leaf is soaked in water and the concoction decanted into the nose of cattle

*Ekebergia capensis *	R & Sb.	H	Gastritis, cough	O	D	In powder form, two teaspoons infusions into one cup of water are taken before meals

*Eleusine floccifolia*	R	L	Rh factor/disease	O	D	It is the same ingredient and preparation method of *A. pulcherrima*

*Eucalyptus camaldulensis*	L	H	Emergency illness	N	F	The leaf is roasted with the seed of *Lepidium sativum* on the heated tool and the smoke is inhaled by adding cool water on the roasted leaf and at the same time the eye is closed

*Eucalyptus globulus*	L	H	Headache	N	F	It is rubbed and its aroma inhaled
	L	H	Common cold	N	F	It is rubbed and its aroma inhaled without calling its name “Nech-Bahirzaf”

*Euphorbia abyssinica*	S	L	Cough (*Kuro*)	N	D	The stem is placed on burning dung and the smoke sniffed to treat Donkey's cough
	La.	H	Ascariasis	O	F	3–5 drops of its latex are homogenized in water with the powder of *Eragrostis tef* and Injera is made that is to be eaten by infected patient
	Fl.	H	Leprosy (*sgadewie*)	D	Fd.	Pounded flower is infused in honey and applied as cream on wound

*Euphorbia platyphyllos*	W	H	Ascariasis	O	D	In powder form, two teaspoons are roasted with equal size of roasted *Hordeum vulgare* in the form of Injera and eaten for three consecutive days
	La.	H	Skin infection (*barle*)	D	F	The latexes are applied as cream on the affected parts only

*Euphorbia polyacantha*	La.	H	Impetigo (*Kunchir*)	D	F	The wound is bled first and then its latex with latex of *Euphorbia tirucalli *is applied as cream
	La.	H	Boils (*Bigungi*)	D	F	Its latex is applied as cream with latex of *C. procera* on the wound part only
	La.	H	Skin cancer (tumor)	D	F	It is the same method of preparation as used in *C. procera*

*Euphorbia tirucalli*	La.	L	Donkey's wart	D	F	The milky latex is pasted alone or with the latex of *E. polycantha* and squeezed leaf of *C. simensis*, after bleeding the wound with blade until recovery

*Euryops pinifolius*	L	H	Stomachache (*kurba*)	O	F	The leaf is chewed and absorbed during feeling pain

*Ferula communis*	R	L	Sterile cow/be fertile	O	D	Roots are dug at seven distinct places; pounded root is eaten with Injera
	R	H	Intestine pain	O	D	Two teaspoonful powders was infused in to glass of water (one cup of tea amount) and drunk

*Ficus palmata*	La.	H	Impetigo (*Kunchir*)	D	F	The wound is bled first and then its latex is applied as cream alone until recovery
	La.	H	Hemorrhoids	D	F	It is the same method and ingredient used in *C. procera*

*Ficus vasta*	L	H	Febrile illness	Op.	F	It is boiled with the leaf of *C. spinarum* and *Zehneria scabra* and then the vapor is inhaled

*Foeniculum vulgare*	L	H	Diuretic (*Shntemat*)	O	D	It is boiled and then the decoction is drunk by adding sugar like tea

*Gossypium hirsutum*	Se.	H	Malaria	O	D	Ground seed is soaked in water with small amount of salt and drunk by a cup of tea

*Grewia ferruginea*	Sb.	L	Leech infestation	N	F	Chopped and pounded stem bark is mixed with water and poured into the nose of cattle
	L	L	Removal of placenta	O	F	It is crushed and pounded with the root of *Verbscum sinaiticum *and decanted for the cattle
	L	H	Eye injury	Op.	F	It is squeezed alone or with *Rumex nervosus* and dropped into eye injured by* Euphorbia* latex

*Guizotia abyssinica*	Se.	H	Dry cough	O	D	It is pounded with seed of *Nigella sativum, A. sativum, *flower of *Thymus schimperi*, and rhizome of* Zingiber officinale *by pestle and mortar and then the concoction is drunk until recovery
	Se.	L	Swelling bull neck	D	D	The seed is chewed and the chewed seed is pitted on the wounded neck of bull

*Hagenia abyssinica*	Sb.	H	Malaria	O	D	It is chopped and powdered with the root of *Silene macrosolen, Phytolacca dodecandra*, and* C. ficifolus *and the leaf of* C. myricoides* and is drunk in a half size cup of coffee
	Se.	H	Tapeworm	O	D	The seed is pounded by pestle and mortar and mixed in ale (before filtration) and then a small amount is eaten in a separate house with one boy; if the risk comes, the “Shiro” stew is taken

*Hibiscus eriospermus*	R	H	Impotency in men	O	Fd.	It is the same method and ingredient used in *C. lanatus*

*Hypoestes forskaolii*	R	H	Stomachache	O	F	The cleaned root is chewed and the liquid absorbed
	L	Hl.	Leech infestation	O/N	F	Pounded leaf is soaked in water and drunk orally for human and decanted nasally for cattle

*Impatiens rothii*	Tu.	H	Idiopathy (*likift*)	D	F	It is the same method as used in *A. camperi *and *B. polystachya *
	Tu.	H	Fire accident	D	Fd.	Chopped and pounded tubular root is pasted on the injured body

*Inula confertiflora*	L	L	Cataract (*mora*)	Op.	F	The young leaf is chewed with the leaf of *A. aspera* and spitted to the affected cattle eye
	L	L	Leech infestation	N	F	It is the same as *E. cymosa*
	R	L	Emaciation	O	Fd.	It is the same method of preparation and ingredient of *C. hypoleuca*

*Jasminum grandiflorum*	Sh.	H	Hemorrhage at birth	V	F	It is crushed with the leaf of *Solanum incanum* and *C. pepo* and applied as cream on the vagina
	L	Hl.	Eye injury	Op.	F	It is the same method of preparation and ingredient of *C. ficifolius*
	L	H	Trachoma (*Aynemaz*)	Op.	F	Its young leaf and leaf of *C. myricoides *and* I. confertiflora *are crushed and dried under sunlight and then powdered to cream the infected part with “cul”

*Juniperus procera*	L	L	Febrile illness	O	F	Crushed and pounded leaf is stirred in water and then decanted for all pack animals
	L	L	Rh factor/disease	O	F	Crushed and pounded leaf is stirred in water and then decanted for cow
	L	L	Leech infestation	N	F	Crushed and pounded leaf is stirred in water and then decanted for cattle

*Justicia schimperiana*	L	L	Body lice	D	F	Its leaf is crushed and pounded and then immersed in water to wash the body of cattle
	L	L	Dysentery	O	F	It is crushed with *Salvia schimperi *and soaked in water with ash for decanting for sheep and goat

*Kalanchoe petitiana*	S	L	Swelling bull neck	D	F	Once bled with blade, chopped and peeled stem twig is tied on injured neck of bull
	Ag.	H	Scabies (*Lifie*)	D	Fd.	It is the same method and ingredient as *D. radicans *

*Lagenaria siceraria*	F	L	Swelling bull neck	D	D	The fruit is roasted, used as container of butter on flame, and the injured neck of bull is rubbed
	L	H	Ringworm	D	F	It is crushed and applied as cream on infected head and other skin parts until recovery

*Laggera tomentosa*	L	H	Stomachache	O	F	The leaf is chewed and the liquid absorbed
	L	Hl.	Leech infestation	O/N	F	It is the same as *H. forskaolii *preparation

*Launaea intybacea*	R	H	Stomachache	O	F	The root is chewed and the liquid absorbed

*Leonotis ocymifolia*	L	L	Bloating	O	F	Crushed and pounded leaf is stirred in water and then decanted for cattle
	L	L	Struck cattle	O	F	Crushed and pounded leaf is stirred in water and then decanted for cattle

*Lepidium sativum*	Se.	L	Bloating (*Nifat*)	O	D	It is the same method and ingredient of *B. nigra*

*Leucas martinicensis*	W	L	Emaciation	O	Fd.	It is the same method of preparation and ingredient of *C.hypoleuca*

*Lippia adoensis*	L	H	Ringworm (*Aguagot*)	D	F	Crushed and pounded leaf is applied as cream with the milk of pregnant cow on the injured part

*Lobelia rhynchopetalum *	R & S	H	Evil eye	O/D	D	It is the same method and ingredient of *A. sativum*
	La.	H	Hemorrhoids/tumor	D	F	It is the same method and ingredient used in *C. procera*

*Malva verticillata*	R	H	Dandruff	D	F	Its root is crushed in water until formation of bubble and then the head is washed
	Sb.	Hl.	Wound (any type)	D	F	Chopped and pounded stem bark is pasted with powdered *Usea *sp. on wound

*Mentha longifolia*	L	L	Leech infestation	N	F	It is pounded and soaked in water and then the concoction decanted in nose of cattle

*Momordica foetida*	W	H	Psychiatric disorder	D	F	The whole parts are pounded and immersed in pot water for three days and body is washed

*Myrica salicifolia*	Sb.	L	Rh factor/disease	O	Fd.	It is the same ingredient and preparation method of *A. pulcherrima*

*Myrsine africana*	F	H	Ascariasis	O	F	Ripe fruit is eaten and traditional beer/ale is drunk in the morning before any meals
	L	H	Tapeworm	O	F	The leaf is pounded and squeezed by adding water and then drinking half a cup of water

*Myrtus communis*	L	H	Itching (*Ekek*)	D	D	The leaf is crushed and powdered with the leaf of *Rhus retinorrhoea* and butter; then the inflamed body is creamed and stayed under sunlight for a few minutes
	Ag.	H	Epidemic	N	D	The crushed aboveground parts are inhaled on hot fire to reduce transmission of disease

*Nicotiana rustica*	L	L	Leech infestation	N	F	The leaf is pounded and soaked in water and then the concoction is decanted into nose of cattle

*Nigella sativa*	Se.	H	Stomachache (*Alsha*)	O	D	The ground seed is boiled with oil and drunk by tea glass
	Se.	H	Dry cough	O	D	It is the same method and ingredient of *G. abyssinica*

*Ocimum lamiifolium*	L	H	Headache; febrile	O/D	F	One cup of squeezed leaf is drunk with coffee orally and the remainder is applied as cream to injured parts

*Olea europaea *ssp. *cuspidata*	S	H	Toothache	D	F	It is similar method to that used in *C. abyssinica*
	S	H	Human's wart	D	F	The bleeding wart is heated with chopped and roasted stem with the sap of *Rumex nervosus*

*Orobanche ramosa*	W	H	Fire accident	D	D	Crushed and roasted parts are powdered and then are applied as cream with butter on injured parts

*Osyris quadripartita*	L	L	Emaciation	O	Fd.	It is the same method of preparation and ingredient of *C. hypoleuca*

*Otostegia fruticosa*	L	H	Diarrhea	O	F	One-half of a cup of coffee of squeezed leaf is drunk, specially for babies

*Otostegia integrifolia*	L	H	Vomiting, nausea, & diarrhea	O	F	The leaf is crushed and squeezed and mixed to root of *Verbena officinalis* in a cup of coffee and then the concoction is drunk
	L	L	Dysentery	O	F	It is crushed and pounded with leaf of *C. hypoleuca *and immersed in water under a new container of* L. siceraria *and then the concoction is decanted for the cattle, specially ox and cow
	Ag.	H	Evil spirit	N	Fd.	The aboveground parts are fumigated on hot fire, specially for the new birth time

*Otostegia tomentosa*	L	H	Ascariasis, diarrhea	O	F	The leaf is pounded and squeezed and then drunk in a cup of tea in the morning before any diet

*Phytolacca dodecandra*	R & L	Hl	Rabies virus	O	D	It is chopped and pounded with the root of *C. macrostachyus *and* Z. scabra *and the powder is mixed with the milk of identical color cow and calf and drunk in half tea glass

*Plectranthus cylindraceus*	L	H	Vomiting & diarrhea	O	F	Crushed and squeezed leaf is mixed in boiled cup of coffee and then the decoction is drunk
	L	H	Emergency (*Wereza*)	O/D	F	It is squeezed and mixed in boiled cup of coffee and then the decoction is drunk and the face is creamed

*Polygala abyssinica*	R	H	Against snake bite	O	F	It is chewed and absorbed before any diet during the starting new year of Ethiopia and did not take any sour taste fruits from the time on wards
	R	H	Evil spirit (*Ayinetila*)	O	Fd.	The cleaned root is chewed and absorbed

*Primula verticillata*	L	Hl.	Cataract (*Mora*)	Op.	F	Leaf is pounded and squeezed by using clean cloth and added to injured or infected eye

*Pteris dentate*	W	H	Evil spirit (*Ejeseb*)	D	Fd.	The powder is tied on the neck and held in the pocket wherever they moved

*Pteris pteridioides*	W	H	Evil spirit (*Ejeseb*)	D	Fd.	It is similar methods as those used in *P. dentata*

*Pulicaria schimperi *	L	H	Wound (infection)	D	F	Pounded leaf is pasted on wounded part

*Rhamnus prinoides*	L	H	Tonsillitis	O/D	Fd.	Pounded and squeezed leaf is taken in a half index finger size of cup and is applied as cream with malt on the center of head by saying “sikel, sikel”

*Rhus glutinosa *ssp. neoglutinosa	L	L	Leech infestation	N	F	The leaf is pounded and stirred in water that contained salt and then the concoction is decanted

*Rhus retinorrhoea*	L	H	Itching (*Ekek*)	D	D	It is the same method and ingredient of *M. communis*

*Rhynchosia minima*	R	L	Weakling bull (*Abayanet*)	O	D	It is chopped and pounded with the root of *Stephania abyssinica *and *Verbena officinalis* which are immersed in a new container of *L. siceraria* for three days and then the concoction is decanted for the bull which is falling during plowing up the farmland

*Ricinus communis*	Sh.	H	Eczema (*Chifie*)	D	Fd.	It is roasted and powdered with the leaf of *C. procera* and mixed with butter and then applied as cream

*Rubus apetalus*	R	H	Evil eye	N	D	Crushed and powdered root is fumigated and the aroma of the smoke at night is smelt

*Rumex abyssinicus*	R	H	Itching (*Ekek*)	D	Fd.	It is crushed and roasted and then powdered root is applied as cream with butter on wound

*Rumex nepalensis*	R	H	Stomachache (*Alsha*)	O	F	The cleaned root is chewed and absorbed alone or with the root of *Solanum incanum*

*Rumex nervosus*	L	H	Eye pain	Op.	F	It is the same as *G. ferruginea*
	S	H	Ringworm (*Agogot*)	D	F	The stem is roasted on hot fire and the bubbles arisen from stem are applied as cream on wound
	S	H	Human's wart	D	F	It is the same as *O. Europaea *ssp.* cuspidata*

*Ruta chalepensis*	F	H	Stomachache (*kurba*)	O	Fd.	The fruit/seed is eaten alone or with the rhizome of *Zingiber officinale* and salt
	F	H	Evil eye (*lebuda*)	D	Fd.	The fruit is chewed and held with the bulb of *A. sativum* and stem of *A. afra*

*Salvia schimperi*	L	L	Dysentery	O	F	It is the same method and ingredient of *J. schimperiana*

*Schinus molle*	F	H	Stomachache (*kurba*)	O	Fd.	The ripe fruit/seed is eaten alone for both prevention and treatment of disease

*Sesamum orientale*	Se.	H	Rh factor/disease	O	D	It is the same method and ingredient used in *A. pulcherrima*

*Sida schimperiana*	R	H	Evil eye	O/D	D	It is the same method and ingredients used in *A. sativum*

*Silene macrosolen*	R	H	Tapeworm	O	Fd.	Crushed and pounded root in half index finger size is drunk by tea glass, and if the risk comes, powder of *Linum usitatissimum* infusion in water is taken as reducing pain

*Solanecio gigas*	L	L	Emaciation	O	F	It is the same method of preparation and ingredient of *C. hypoleuca*

*Solanum anguivi*	F	H	Birth control	O	F	It is the same method of preparation and ingredient used in *C. ficifolus*
	F	H	Itching (*Ekek*)	D	F	The fruit is pounded with pestle and mortar and then applied as cream with butter on wound

*Solanum marginatum*	F & Se.	L	Cough (*Chifra*)	N	F	Its fruit is pierced and its fluid and seed are collected in a cup to be decanted nasally
	F & Se.	H	Rabies virus	O	F	Its fluid is collected with squeezed leaf of *P. dodecandra* and then drunk with cup of tea
	F	L	Evil eye	O	F	Roasted and pierced ripe fruit can be eaten with straw or hay fodders

*Solanum incanum*	L	Hl.	Bleeding (epistaxis)	D	F	Its leaf is crushed and tied to bleeding nose alone or with the leaf of *A. aspera *and* R. nervosus*
	R	H	Stomachache	O	F	It is the same method and ingredient used in *R. nepalensis*

*Solanum nigrum*	L	H	Gastritis	O	F	The washed or cleaned raw leaf before any meals is chewed

*Stephania abyssinica*	R	H	Against snake bite	O	F	It is the same as *P. abyssinica* except that there is no restriction on taking any sour taste fruits

*Striga hermonthica*	W	L	Poisoned insects	O	Fd.	It is the same method and ingredients used in *C. macrostachyus*

*Thymus schimperi*	F	H	Dry cough	O	D	It is the same method and ingredients used in *G. abyssinica*
	L	H	Hypertension	O	Fd.	The crushed or the normal leaf is boiled and then taken like a tea with a tea glass

*Tragia brevipes*	W	H	Impotency (in men)	O	Fd.	It is the same method and ingredient used in *C. lanatus*

*Urtica simensis*	L	H	Night blindness	D/N	F	The leaf is boiled in hot water and its aroma fumigated three times by opening the lid
	Rh.	H	Abortion	V	F	It is peeled with blade; three half little finger size rhizomes are inserted in the vagina
	L	H	Eye injury	Op	F	Pounded and squeezed leaf is dropped on the injured eye by insertion of bad materials

*Verbena officinalis*	R	H	Stomachache/nausea	O	F	The liquid is chewed and sucked and the residue is spitted
	R	L	Weakling bull	O	D	It is the same method and ingredient used in *R. minima*

*Verbscum sinaiticum*	R	L	Struck cattle (*Betir*)	O	Fd.	One water cup of pounded root in the water is decanted alone or with *E. hispidus*
	R	L	Febrile illness	O	Fd.	Crushed and pounded root is soaked in water and poured orally for all livestock
	R	H	Evil eye	O/N	D	It is the same method and ingredient used in *A. sativum*
	R	H	Psychiatric disorder	O	Fd.	It is crushed alone or with *C. ficifolus* and then half index finger size by a tea glass is drunk

*Vernonia leopoldii*	L	H	Wound, bleeding	D	F	The leaf is crushed and pounded and then tied on the injured part

*Vernonia schimperi*	R	H	Febrile illness	D/N	Fd.	Crushed and pounded leaf is stirred in water until bubbles are formed and then all parts are creamed except chest and the dried root is fumigated on hot fire and sniffed

*Vicia faba*	Se.	H	Boils	D	D	It is ground and soaked in water and pasted on wound and then heated by dung of jackass
	Se.	H	Ascariasis	O	D	It is the same method and ingredient used in either *B. abyssinica* or *C. macrostachyus*

*Withania somnifera*	R	H	Evil eye/spirit	D/N	D	It is the same method and ingredient used in *A. sativum*

*Zehneria scabra*	L	H	Jaundice	O	F	The leaf is pounded and squeezed and then drunk in half a cup of tea
	R	Hl.	Rabies virus	O	D	It is the same method and ingredient used in *P. dodecandra*
	Ag.	H	Febrile illness	N	F	It is the same method and ingredient used in *F. vasta*

*Zingiber officinale*	Rh.	H	Stomachache	O	Fd.	It is the same method and ingredient used in *R. chalepensis*

For authorities to scientific names of each species, see Appendix 1.

**Table 2:** Frequency of plant parts used and route of administration of remedy from TMPs.

Plants parts	Frequency	Percentage	Route of administration	Frequency	Percentage
Leaf only	75	32.6	Oral	101	43.9
Root only	50	21.7	Dermal	66	28.7
Fruit only	16	6.9	Nasal	26	11.3
Seed only	15	6.5	Optical	11	4.8
Whole plant	15	6.5	Oral and dermal	10	4.3
Latex only	12	5.2	Dermal and nasal	8	3.5
Stem only	8	3.5	Oral and nasal	5	2.2
Aboveground parts	7	3.0	Vaginal	2	0.9
Bulb	6	2.6	Anal	1	0.4
Root and stem	5	2.2	Total	**230**	** 100.0**
Stem bark only	5	2.2			
Rhizome only	3	1.3			
Root and stem bark	3	1.3			
Flower only	2	0.9			
Fruit and seed	2	0.9			
Shoot only	2	0.9			
Tuber	2	0.9			
Leaf and stem	1	0.4			
Root and leaf	1	0.4			
Total	**230**	**100.0**			

**Table 3:** Informant consensus on commonly known TMP species.

Scientific name	Total informants' agreement	Percentage	Rank
*Ocimum lamiifolium*	66	91.7	1st
*Zehneria scabra*	61	84.7	2nd
*Carduus schimperi*	56	77.8	3rd
*Achyranthes aspera*	42	58.3	4th
*Schinus molle*	37	51.4	5th
*Allium sativum*	35	48.6	6th
*Echinops hispidus*	30	41.7	7th
*Cucumis ficifolius*	25	34.7	8th
*Aloe pulcherrima*	21	29.2	9th
*Withania somnifera*	20	27.8	10th

**Table 4:** Preference ranking of TMPs against febrile illness.

Plant species that treat febrile illness	Key informants (coded K_1_ to K_9_) with the ranks they gave										
	K_1_	K_2_	K_3_	K_4_	K_5_	K_6_	K_7_	K_8_	K_9_	Total	Rank
*Carduus schimperi*	4	1	2	5	4	3	5	3	1	28	3rd
*Echinops hispidus *	5	4	5	1	2	2	3	2	3	27	4th
*Ocimum lamiifolium*	3	5	4	3	5	4	2	4	5	35	1st
*Vernonia schimperi*	1	3	1	2	1	1	4	1	4	18	5th
* Zehneria scabra*	2	2	3	4	3	5	1	5	5	30	2nd

**Table 5:** Results of paired comparison for five TMPs used for treating stomachache.

Plant species that treat stomachache	Key informants (Coded K_1_ to K_9_) with the ranks they gave										
	K_1_	K_2_	K_3_	K_4_	K_5_	K_6_	K_7_	K_8_	K_9_	Score	Rank
*Rumex nepalensis*	0	2	1	0	3	1	4	0	0	11	4th
*Ruta chalepensis*	2	1	3	2	4	0	0	2	1	15	3rd
*Schinus molle*	4	3	2	4	2	3	3	3	4	28	1st
*Solanum incanum*	1	0	0	1	0	2	2	1	2	9	5th
*Verbena officinalis*	3	4	4	3	1	4	1	4	3	27	2nd

**Table 6:** Informant consensus factor for the given disease category.

Category	N. spp.	Nur.	ICF
Respiratory, febrile illness, and throat infection (common cold, influenza, dry cough, tonsillitis, and sunstroke)	19	203	0.91

Internal parasites and gastrointestinal disorder (rabies virus, jaundice, stomachache, bloating, vomiting and nausea, appetite loss, malaria, diarrhea and dysentery, tapeworm, ascariasis, giardiasis, and amoebiasis, leech infestation, and heartburn)	46	248	0.82

Dermatological disorders/infections (swelling, wound, dandruff, ring worm, leprosy, eczema, itching, wart, boils, impetigo, and idiopathy)	34	178	0.81

Psychiatric disorder and birth problems (headache, toothache, blood pressure, abortion, Rh factor, retained placenta, hemorrhage at birth, and infertility)	19	68	0.73

Emaciation and weakling for livestock and epidemic for humans	9	30	0.72

Evil eye, evil spirit, and emergency (trauma)	24	59	0.60

External injury and poisoning parasites (eye problems, cataract, bone fracture, circumcision, bleeding, snake bite, struck, fire accident, and body lice)	25	48	0.49

Genitourinary problems (gonorrhea and diuretic and sexual impotency)	5	8	0.43

**Table 7:** Results of direct matrix ranking of multipurpose TMPs.

Main uses	*Carissa spinarum*	*Olea europaea *ssp. *cuspidata*	*Rhus glutinosa *ssp. *neoglutinosa*	*Ficus palmata*	*Ficus vasta*	*Rubus apetalus*
Firewood	5	5	5	5	5	5
Medicine	5	5	3	5	3	3
Construction	4	5	5	4	5	1
Fodder	4	5	5	0	0	3
Edible fruit	5	0	3	5	5	5
Total	23	20	21	19	18	17
Rank	1st	3rd	2nd	4th	5th	6th

**Table 8:** Results of direct matrix ranking of factors threatening to TMPs.

Threats	Key informants (coded K_1_ to K_9_) with the total scores and rank										
	K_1_	K_2_	K_3_	K_4_	K_5_	K_6_	K_7_	K_8_	K_9_	Total	Rank
Agricultural expansion	6	6	5	5	6	5	4	6	6	49	1st
Mining of opals	4	3	5	3	5	4	5	4	3	33	5th
Overgrazing	4	5	4	4	6	3	3	5	4	38	4th
Farm tools and construction	6	6	4	3	3	6	4	3	6	41	3rd
Drought	3	4	3	4	2	5	3	2	5	31	6th
Firewood and charcoal	5	5	6	5	5	6	6	5	4	47	2nd

**Table 9:** Priority ranking values (based on their degree of scarcity in the wild) for five selected TMPs.

Threatened medicinal plant species	Key informants (coded K_1_ to K_6_) with the total scores and rank										
	K_1_	K_2_	K_3_	K_4_	K_5_	K_6_	K_7_	K_8_	K_9_	Total	Rank
*Olea europaea *ssp. *cuspidata*	4	3	4	5	4	3	1	5	2	31	2nd
*Croton macrostachyus*	2	1	1	2	1	5	4	3	4	23	4th
*Juniperus procera*	3	5	3	3	5	1	5	2	1	28	3rd
*Aloe pulcherrima*	1	2	2	1	3	4	2	1	5	21	5th
*Withania somnifera*	5	4	5	4	2	2	3	4	3	32	1st

**Table 10:** LK of people on landscapes and soil types.

Landscape classification		Soil classification	
Emic classification	Etic classification	Emic classification	Etic classification
WOTAGEBA	Undulated land	KEYATIE	Red soil
TERRAR	Mountain	WALKA	Black soil
MEDA/REGATA	Plain	BUNAMA	Dark brown
GODGUADAMESK	Valley	ASE	Mixed soil
GEFET/GEDEL	Outcrop land	NECHATE	Silt/ashy soil
TEDAFAT/KONTER	Hilly/steep slope	KOSIE	Dung wastes

**Table 11:** Plant community types and characteristic plant species.

Number	Plant community type and elevation	Characteristic species	Taxa known to be endemic or near endemic to Ethiopia	Spp.
I	*Becium grandiflorum *and *Rumex nervosus* (2502 and 2738 m); ASHA (restored vegetation)	*B. grandiflorum* ^+^ *, R. nervosus*	*B. grandiflorum* ^++^ *, Laggera tomentosa* ^++^	35

II	Riverine vegetation (altitude varies); WENZ DAR	*Ehretia cymosa, Cordia africana*, and* Ficus *spp.	*Urtica simensis* ^*+*^ *, Impatiens rothii* ^++^	5

III	*Otostegia integrifolia *and* Dodonaea angustifolia* (1802–2500 m); QUTQUATO (shrub)	*O. integrifolia, D. angustifolia *	*Vernonia leopoldii* ^+^	32

IV	Farmland and monastery plant community type (altitude varies); YERSHAGOT and GEDAM	*Olea europaea *ssp. *cuspidata*, *Juniperusprocera, *	*Erucastrum abyssinicum* ^++^,* Rhus glutinosa *ssp. *neoglutinosa* ^+^	20

V	Homegarden plant community type (altitude varies); YEGUARO ATIKLT	*Rhamnus prinoides, Calpurnia aurea, *and *Hagenia abyssinica *	*Aloe pulcherrima* ^+^ *, Solanecio gigas* ^+^	24

VI	*Conyza hypoleuca *and *Clutia abyssinica *dominated community type (2814–3253 m); MUSH (bush)	*C. hypoleuca, C. abyssinica *	*Inula confertiflora* ^+^ *, Solanum marginatum* ^++^ *, Otostegia tomentosa* ssp. steudneri^+^, * Lippia adoensis* ^++^, and * Kalanchoe petitiana* ^++^	9

VII	*Eucalyptus* plantation dominated community type (3200–3253 m); DEN/KILKIL (jungle)	*E. globulus, E. camaldulensis, *and *Carduus schimperi*	*Thymus schimperi* ^++^	4

VIII	*Lobelia rhynchopetalum *and *Euryops pinifolius *dominated community type (3539–3702 m); CHEKA (forest)	*L. rhynchopetalum* ^+^ *, E. pinifolius* ^+^	*L. rhynchopetalum* ^+^ *, E. pinifolius* ^+^ *, Sedum mooneyi* ^+^, and *Primula verticillata *ssp. *simensis* ^++^	4

	Total			**133**

^+^Taxa which are endemic to Ethiopia while ^++^near endemic taxa are those shared with Eritrea.

**Table 12:** Mode of TMP knowledge transfer from traditional healers to others.

Means of knowledge transfer	Number of traditional healers	%
Selected family members (verbal and/or observation)		
Males only	9	50.0
For both females and males	6	33.4
Other members of societies (verbal and/or observation)		
Best relatives/peers	2	11.1
Anybody who seeks to learn the knowledge of healers	1	5.5
Total	**18**	**100.0**

**Table 13:** Variation of LK on some TMP names.

Number	Scientific name	Different local names among three groups of informants		
		Key informants	Permanent residents	Returnees
1	*Aeonium leucoblepharum*	Gimdo	Yetota-Kita	—
2	*Asparagus africanus*	Yeset-kest	Kestencha	Betin
3	*Carduus schimperi*	Dendero	Yemidir Koshele	Yedega Koshele
4	*Carthamus lanatus*	Yesetaf	Koshele	—
5	*Ceratostigma abyssinicum*	Key-telenji	Key-telenji	—
6	*Chenopodium schraderianum*	Sinin	Sinign	—
7	*Cistanche tubulosa*	Dechmerch	Yesatmedanit	—
8	*Croton macrostachyus*	Bisana	Bisana/Mekenisa	Mekenisa
9	*Cyathula uncinulata*	Kuno	Kugno	—
10	*Cynoglossum coeruleum*	Yemogn-fikir	Hulu-zemede	Chigogot
11	*Cyphostemma adenocaula*	Milas-golgul	Aserkush	Hareg
12	*Datura stramonium*	Astenagr	Banjie	—
13	*Dyschoriste radicans*	Yesheftmedanit	Yesheftmedanit	—
14	*Ekebergia capensis*	—	—	—
15	*Euphorbia polyacantha*	Sete-qulqual	Yeberha-qulqual	Qulqual
16	*Justicia schimperiana*	Simiza/Sensel	Sensel	Sensel
17	*Kalanchoe petitiana*	Endehulla	Endehulla/Fitfita	—
18	*Laggera tomentosa*	Keskessie	Alashume	Alashume
19	*Launaea intybacea *	Demastefi	Yewushamilas	Yewushamilas
20	*Lippia adoensis*	Kessie	Ayib-kessie	—
21	*Myrsine africana*	Kechemo	Kerchemo	—
22	*Nicotiana rustica *	Yabesh-Tinbaho	Tinbaho/Atiya	Tinbaho
23	*Orobanche ramosa*	—	Yesatmedanit	—
24	*Plectranthus cylindraceus*	—	Yewerezamedanit	—
25	*Polygala abyssinica *	Etse-libona	Yebabmedanit/Kibegolgu	—
26	*Pulicaria schimperi *	—	—	—
27	*Ricinus communis *	Gullo	Agullo	Agullo
28	*Rumex abyssinicus *	Mekmeko	Embari-kolla	Embari-kolla
29	*Solanum incanum *	Yedi	Embuay	Embuay
30	*Solanum nigrum *	Tikur-awut	Tikur-awut	Awut
31	*Stephania abyssinica*	Etse-eyesus	Yeayit-hareg	Hareg
32	*Pteris pteridioides *	Etse-anbessa	Ems-anketkit	—
33	*Verbscum sinaiticum *	Etse-debtera	Ketetina	Ketetina
34	*Vernonia schimperi *	—	Yemichmedanit	—
35	*Zehneria scabra *	Etse-sabek	Aregresa	Hareg
