# Supplementary material for: The Status of Ethnobotanical Knowledge of Medicinal Plants and the Impacts of Resettlement in Delanta, Northwestern Wello, Northern Ethiopia
Source: Evid Based Complement Alternat Med. 2016 Jan 13;2016:5060247. doi: 10.1155/2016/5060247 (PMC4737471; doi:10.1155/2016/5060247)
Supplement: Supplementary file 1 — A total of 133TMP species belonged to 116 genera and 57 families were documented in Delanta. Those species were collected from different habitats where they are varied in altitudinal ranges from 1802 to 3702 m a.s.l., growth habits and species abundance distribution. The botanical names of all species with authorities and their local names are given in Appendix 1. [file 5060247.f1.pdf]

Appendix 1: List of total MP species with local names, habits and habitats collected in the study area

Description of data: Ha-Habit (S= Shrub H=Herb T=Tree); Hab-Habitat (Hg=Homegarden, Hgw=Homegarden & Wild, Fl=Farmland, Ev=Enclosed vegetation, Evu=Enclosed and unenclosed vegetation, Al=All); + indicates - endemic to Ethiopia and/or shared with Eritrea, * not listed as multiuse; ***cultivated.

No.	**Scientific name**	**Local name (Amharic)**	**Family**	**Ha.**	**Hab.**	**Spp. Abu**	**Geographical location**	**Coll.No**
1	*Achyranthes aspera* L.	Telenj	Amaranthaceae	H	Al	Spa	2653m; 11^{0}40.177’N, 039^{0}19.813’E	MM005
2	*Aeonium leocoblepharum* A.Rich.	Yetota-kita	Crassulaceae	H	Evu	Rare	2578m; 11^{0}40.165’N, 039^{0}19.969’E	MM112
3	*Ajuga integrifolia** Ham.-Buch.	Tut-astil	Lamiaceae	H	Evu	Rare	2664m; 11^{0}40.180’N, 039^{0}19.811’E	MM092
4	*Allium cepa* L.***	Key Shinkurt	Alliaceae	H	Hg	Com	2665m; 11^{0}40.157’N, 039^{0}19.803’E	MM117
5	*Allium sativum* L.***	Nech Shinkurt	Alliaceae	H	Hg	Spa	2667m; 11^{0}40.047’N, 039^{0}19.809’E	MM044
6	*Aloe camperi* Schweinf.	Wende-eret	Aloaceae	S	Evu	Com	2625m; 11^{0}39.056’N, 039^{0}19.478’E	MM124
7	*Aloe pulcherrima*$^{+}$ Gilbert & Sebsebe (ined.)	Set-eret	Aloaceae	S	Hgw	Rare	2667m; 11^{0}40.047’N, 039^{0}19.809’E	MM43
8	*Artemisia abyssinica** Sch.Bip.ex A.Rich.	Chikugn	Asteraceae	H	Al	Com	2667m; 11^{0}40.047’N, 039^{0}19.809’E	MM042
9	*Arundo donax* L.	Shenbeqo	Poaceae	H	Ev	Spa	2181m; 11^{0}42.527’N, 039^{0}20.960’E	MM158
10	*Asparagus africanus* Lam.	Kestenicha	Asparagaceae	S	Evu	Rare	2651m; 11^{0}38.147’N, 039^{0}19.830’E	MM23
11	*Bersama abyssinica* Fresen.	Azamir	Melianthaceae	S	Evu	Rare	2584m; 11^{0}40.074’N, 039^{0}19.594’E	MM53
12	*Brassica nigra* (L.) Koch in Rohling***	Senafich	Brassicaceae	H	Fl	Spa	2675m; 11^{0}40.175’N, 039^{0}19.813’E	MM039
13	*Buddleja polystachya* Fresen.	Amfar	Loganiaceae	S	Evu	Rare	2530m; 11^{0}40.046’N, 039^{0}19.754’E	MM017
14	*Caesalpina decapetala (Roth.)*	Kentefa	Fabaceae	Cl	Evu	Rare	2577m; 11^{0}39.612’N, 039^{0}19.556’E	MM125

	Alston							
15	*Calotropis procera** (Ait) Ait.f.	Tobbiya	Asclepiadaceae	S	Evu	Rare	2205m; 11^{0}38.528’N, 039^{0}19.813’E	MM079
16	*Calpurnia aurea* (Ait.) Benth.	Digita	Fabaceae	S	Hgw	Spa	2528m; 11^{0}40.041’N, 039^{0}19.700’E	MM018
17	*Capparis tomentosa* Lam.	Gimero	Capparidaceae	S	Evu	Rare	2093m; 11^{0}38.342’N, 039^{0}20.274’E	MM073
18	*Capsicum annuum* L.***	Kariya	Solanaceae	H	Hg	Spa	2193m; 11^{0}41.174’N, 039^{0}20.908’E	MM131
19	*Carduus schimperi* Sch. Bip.ex A.Rich.	Yemidr Koshele	Asteraceae	H	Evu	Rare	3202m; 11^{0}40.551’N, 039^{0}15. 534’E	MM103
20	*Carissa spinarum* L.	Agam	Apocynaceae	S	Evu	Spa	2392m; 11^{0}38.818’N, 039^{0}19.454’E	MM021
21	*Carthamus lanatus* L.	Koshele	Asteraceae	H	Fl	Spa	2586m; 11^{0}40.177’N, 039^{0}19.960’E	MM136
22	*Catha edulis* (Vahl.) Forssk.ex Endl.***	Chat	Celastraceae	T	Hg	Spa	2668m; 11^{0}40.174’N, 039^{0}19.812’E	MM091
23	*Ceratostigma abyssinicum** (Hochst.) Schweinf. & Asch.	Key Telenj	Plumbaginaceae	S	Evu	Spa	2530m; 11^{0}40.046’N, 039^{0}19.754’E	MM016
24	*Chenopodium schraderianum* Schult.	Sinign	Chenopodiaceae	H	Fl	Spa	2584m; 11^{0}40.074’N, 039^{0}19.594’E	MM050
25	*Cistanche tubulosa** (Schenk) Hook.f.	Yesatmedanit	Orobanchaceae	H	Fl	Rare	2205m; 11^{0}38.528’N, 039^{0}19.813’E	MM165
26	*Citrus aurantifolia* (Christm.) Swingle***	Lomie	Rutaceae	S	Hg	Spa	1994m; 11^{0}38.038’N, 039^{0}20.573’E	MM076
27	*Clematis semensis* Fresen.	Azo-hareg	Ranunculaceae	Cl	Evu	Spa	2584m; 11^{0}40.074’N, 039^{0}19.594’E	MM054
28	*Clerodendrum myricoides* (Hochst.) Vatke.	Missiroch	Lamiaceae	S	Ev	Spa	2392m; 11^{0}38.818’N, 039^{0}19.454’E	MM045
29	*Clutia abyssinica* Jaub. &Spach.	Fiyel-afege	Euphorbiaceae	S	Evu	Com	2997m; 11^{0}40.404’N, 039^{0}19.086’E	MM110
30	*Colutea abyssinica* Kunth & Bouche	Duaduate	Fabaceae	S	Ev	Rare	3200m; 11^{0}40.520’N, 039^{0}16.799’E	MM104
31	*Combretum molle* R. Br.ex G. Don.	Aballo	Combretaceae	T	Evu	Rare	2097m; 11^{0}38.259’N, 039^{0}20.309’E	MM081
32	*Conyza hypoleuca* A. Rich.	Nechillo	Asteraceae	S	Evu	Com	2814m; 11^{0}36.829’N, 039^{0}13.637’E	MM161
33	*Coriandrum sativum* L.***	Dimblal	Apiaceae	H	Hg	Com	2664m; 11^{0}40.180’N, 039^{0}19.811’E	MM093
34	*Croton macrostachyus* Del.	Mekenisa/Biss ana	Euphorbiaceae	T	Hgw	Rare	2485m; 11^{0}40.011’N, 039^{0}19.898’E	MM032

35	*Cucumis ficifolus* A.Rich	Yemdir-embuay	Cucurbitaceae	H	Al	Spa	2656m; 11^{0}38.117’N, 039^{0}19.806’E	MM22
36	*Cucurbita pepo* L.***	Duba	Cucurbitaceae	H	Hg	Spa	2669m; 11^{0}40.167’N, 039^{0}19.811’E	MM115
37	*Cyathula uncinulata** (Schrad.) Schinz.	Kuno/Kugno	Amaranthaceae	H	Evu	Com	2584m; 11^{0}40.074’N, 039^{0}19.594’E	MM052
38	*Cynoglossum coeruleum* Hochst. ex A.DC. in DC. ssp. Johnstonii	Hulu-zemede	Boraginaceae	H	Fl	Com	2672m; 11^{0}39.432’N, 039^{0}18.692’E	MM048
39	*Cyphostemma adenocaule** (Steud.ex A. Rich) Desc. ex Wild & Dr.	Aserkush	Vitaceae	Cl	Evu	Com	2205m; 11^{0}38.528’N, 039^{0}19.813’E	MM082
40	*Datura stramonium** L.	Banjie	Solanaceae	H	Hg	Com	2531m; 11^{0}40.047’N, 039^{0}19.755’E	MM014
41	*Debregeasia saeneb* (Forssk.) Hepper & Wood	Dorofes	Urticaceae	S	Evu	Com	2596m; 11^{0}41.975’N, 039^{0}20.684’E	MM150
42	*Dodonaea angustifolia* L.f.	Kitkita	Sapindaceae	S	Evu	Com	2500m; 11^{0}40.965’N, 039^{0}19.850’E	MM030
43	*Dyschobriste radicans* (A.Rich.) Nees	Yesheftmedan it	Acanthaceae	H	Evu	Spa	2522m; 11^{0}38.041’N, 039^{0}19.717’E	MM029
44	*Echinops hispidus** Fresen.	Kebercho	Asteraceae	H	Fl	Rare	2680m; 11^{0}40.218’N, 039^{0}19.693’E	MM058
45	*Ehretia cymosa* Thonn.	Wulaga	Boraginaceae	S	Ev	Rare	2069m; 11^{0}40.532’N, 039^{0}21.077’E	MM097
46	*Ekebergia capensis* Sparrm.	------	Meliaceae	T	Ev	Spa	2584m; 11^{0}40.074’N, 039^{0}19.594’E	MM168
47	*Eleusine floccifolia*	Akrma	Poaceae	H	Hg	Com	2841m; 11^{0}42.527’N, 039^{0}20.960’E	MM164
48	*Eucalyptus camaldulensis* Dehnh.***	Key Bahrzaf	Myrtaceae	T	Ev	Spa	2539m; 11^{0}36.827’N, 039^{0}13.688’E	MM143
49	*Eucalyptus globulus* Labill.***	Nech Bahrzaf	Myrtaceae	T	Ev	Com	3253m; 11^{0}40.171’N, 039^{0}16.756’E	MM108
50	*Euphorbia abyssinica* Gmelin.	Qulqual	Euphorbiaceae	T	Hgw	Com	2506m; 11^{0}39.993’N, 039^{0}20.053’E	MM094
51	*Euphorbia platyphyllos** L.	Anterfa	Euphorbiaceae	H	Fl	Com	1802m; 11^{0}36.627’N, 039^{0}13.588’E	MM154
52	*Euphorbia polyacantha** Boiss.	Yeberha-qulqual	Euphorbiaceae	S	Evu	Rare	2299m; 11^{0}38.086’N, 039^{0}19.893’E	MM089
53	*Euphorbia tirucalli* L.	Denbekinchbt	Euphorbiaceae	S	Hg	Spa	2007m; 11^{0}40.853’N, 039^{0}19.767’E	MM090
54	*Euryops pinifolius*[+] A. Rich.	Cherenfe	Asteraceae	H	Evu	Spa	3676m; 11^{0}44.819’N, 039^{0}26.415’E	MM149

55	*Ferula communis* L.	Dog	Apiaceae	H	Evu	Com	2959m; 11^{0}39.984’N, 039^{0}19.066’E	MM060
56	*Ficus palmata* Forssk.	Beles	Moraceae	S	Evu	Spa	2572m; 11^{0}40.054’N, 039^{0}19.583’E	MM049
57	*Ficus vasta* Forssk.	Warka	Moraceae	T	Evu	Spa	2343m; 11^{0}38.727’N, 039^{0}19.443’E	MM071
58	*Foeniculum vulgare** Mill.***	Ensilal	Apiaceae	H	Fl	Rare	2600m; 11^{0}39.277’N, 039^{0}19.841’E	MM085
59	*Gossypium hirsutum* L.***	Tit	Malvaceae	S	Hg	Rare	2244m; 11^{0}38.619’N, 039^{0}19.781’E	MM084
60	*Grewia ferruginea* Hochst.ex A.Rich.	Lenquata	Tiliaceae	S	Ev	Spa	2193m; 11^{0}41.174’N, 039^{0}20.908’E	MM132
61	*Guizotia abyssinica* (L.f.) Cass.***	Nug	Asteraceae	H	Fl	Com	2665m; 11^{0}40.178’N, 039^{0}19.812’E	MM128
62	*Hagenia abyssinica* (Bruce) J.F. Gmel.	Kosso	Rosaceae	T	Hg	Rare	3206m; 11^{0}40.250’N, 039^{0}16.915’E	MM140
63	*Hibiscus eriospermus* Hochst.ex Cuf.	Tija-chenger	Malvaceae	S	Ev	Spa	2387m; 11^{0}40.949’N, 039^{0}21.287’E	MM098
64	*Hypoestes forskaolii (Vahl)* R.Br.	Key-matebia	Acanthaceae	H	Evu	Com	2653m; 11^{0}38.432’N, 039^{0}18.692’E	MM087
65	*Impatiens rothii*[+] Hook.f.	Gushirt	Balsaminaceae	H	Evu	Rare	2632m; 11^{0}40.166’N, 039^{0}19.898’E	MM138
66	*Inula confertiflora*[+] A. Rich.	Weynagift	Asteraceae	S	Hgw	Com	2997m; 11^{0}40.404’N, 039^{0}19.086’E	MM109
67	*Jasminum grandiflorum* L.	Tembelel	Oleaceae	S	Hgw	Spa	2662m; 11^{0}38.432’N, 039^{0}18.693’E	MM006
68	*Juniperus procera* Hochst. ex A. Engl.	Tid	Cuppressaceae	T	Ev	Rare	2661m; 11^{0}40.161’N, 039^{0}19.815’E	MM127
69	*Justicia schimperiana* (Hochst.ex Nees) T. Anders	Sensel	Acanthaceae	S	Hgw	Spa	2700m; 11^{0}40.125’N, 039^{0}19.770’E	MM306
70	*Kalanchoe petitiana*[+] A.Rich.	Endehulla/Fitfita	Crassulaceae	H	Evu	Com	2815m; 11^{0}43.263’N, 039^{0}22.435’E	MM147
71	*Lagenaria siceraria* (Molina) Standl.***	Kil	Cucurbitaceae	H	Hg	Com	2669m; 11^{0}40.157’N, 039^{0}19.803’E	MM116
72	*Laggera tomentosa*[+] (Sch. Bip.ex A. Rich.) Oliv. & Hiern.	Alashume	Asteraceae	H	Al	Com	2632m; 11^{0}40.166’N, 039^{0}19.898’E	MM137
73	*Launaea* intybacea (Jacq.) Beauv.	Yewushamlas	Asteraceae	H	Fl	Com	2815m; 11^{0}43.263’N, 039^{0}22.435’E	MM145
74	*Leonotis ocymifolia* (Burm.f.) Iwarsson	Ferszeng	Lamiaceae	S	Hgw	Rare	2997m; 11^{0}40.404’N, 039^{0}19.086’E	MM111
75	*Lepidium sativum* L.***	Feto	Brassicaceae	H	Hg	Com	2659m; 11^{0}40.174’N, 039^{0}19.811’E	MM010

76	*Leucas martinicensis** (Jacq.) R.Br.	Raskemir	Lamiaceae	H	Fl	Com	2662m; 11^{0}40.172’N, 039^{0}19.823’E	MM067
77	*Lippia adoensis*$^+$ Hochst. Ex Walp.	Ayib-kessie	Verbenaceae	S	Evu	Com	2814m; 11^{0}36.829’N, 039^{0}13.637’E	MM139
78	*Lobelia rhynchopetalum*$^+$ *Hemsl.*	Jebera	Lobiliaceae	H	Evu	Rare	3702m; 11^{0}44.968’N, 039^{0}26.505’E	MM152
79	*Malva verticillata* L.	Lut	Malvaceae	H	Fl	Com	2679m; 11^{0}40.175’N, 039^{0}19.812’E	MM114
80	*Mentha longifolia* (L.) Huds.	Shall	Lamiaceae	H	Evu	Com	2181m; 11^{0}40.604’N, 039^{0}20.817’E	MM156
81	*Momordica foetida** Schumach.	Yekura-hareg	Cucurbitaceae	Cl	Evu	Spa	2585m; 11^{0}43.535’N, 039^{0}23.063’E	MM121
82	*Myrica salicifolia* Hochst.ex A.Rich.	Shinet	Myricaceae	T	Evu	Rare	2596m; 11^{0}41.972’N, 039^{0}20.683’E	MM148
83	*Myrsine africana* L.	Kerchemo	Myrsinaceae	S	Ev	Spa	2621m; 11^{0}39.852’N, 039^{0}19.352’E	MM126
84	*Myrtus communis* L.***	Ades	Myrtaceae	S	Hg	Rare	2662m; 11^{0}38.432’N, 039^{0}18.693’E	MM162
85	*Nicotiana rustica* L.***	Tinbaho/Atiya	Solanaceae	H	Hg	Rare	2902m; 11^{0}38.555’N, 039^{0}18.847’E	MM122
86	*Nigella sativa* L.***	Tikur Azmud	Ranunculaceae	H	Hg	Rare	2004m; 11^{0}38.065’N, 039^{0}20.557’E	MM080
87	*Ocimum lamiifolium* Hochst.ex Benth.	Damakessie	Lamiaceae	S	Hgw	Rare	2641m; 11^{0}40.123’N, 039^{0}19.765’E	MM011
88	*Olea europaea* ssp.cuspidata (Wall.ex G. Don) C.f.	Weyira	Oleaceae	T	Hgw	Rare	2672m; 11^{0}39.432’N, 039^{0}18.692’E	MM047
89	*Orobanche ramosa** L.	Yesatmedanit	Orobanchaceae	H	Fl	Rare	2524m; 11^{0}40.041’N, 039^{0}19.876’E	MM035
90	*Osyris quadripartita* Decn.	Keret	Santalaceae	S	Ev	Rare	2522m; 11^{0}38.041’N, 039^{0}19.717’E	MM066
91	*Otostegia fruticosa* (Forssk.) Schweinf. ex Penzig	Geram-tinjut	Lamiaceae	S	Ev	Spa	2575m; 11^{0}40.148’N, 039^{0}19.959’E	MM070
92	*Otostegia integrifolia* Benth.	Tinjut	Lamiaceae	S	Evu	Com	2392m; 11^{0}38.818’N, 039^{0}19.454’E	MM019
93	*Otostegia tomentosa*$^+$ (Chiov.) Sebald.	Tarista	Lamiaceae	S	Evu	Spa	2995m; 11^{0}40.447’N, 039^{0}19.101’E	MM135
94	*Phytolacca dodecandra* L’Herit	Endod	Phytolaccaceae	S	Hg	Com	2640m; 11^{0}38.555’N, 039^{0}18.847’E	MM015
95	*Plectranthus cylindraceus* Hochst.ex Benth.	Yewereza	Lamiaceae	H	Evu	Spa	2654m; 11^{0}40.115’N, 039^{0}19.776’E	MM012
96	*Polygala abyssinica* Fresen.	Yebabmedanit	Polygalaceae	H	Ev	Rare	2530m; 11^{0}40.174’N, 039^{0}19.756’E	MM040

97	*Primula verticilata** Forssk. ssp. simensis^{+}* (Hochst.) W.W.Sm. & Forrest	Ayin-abirra	Primulaceae	H	Evu	Rare	3539m; 11^{0}44.500’N, 039^{0}26.133’E	MM146
98	*Pteris dentate** Forssk.	Emis-anketkit	Pteridaceae	H	Evu	Com	2718m;11^{0}36.776’N, 039^{0}13.814’E	MM167
99	*Pteris pteridioides** (Hook.) Ballard.	Etse-anbessa	Pteridaceae	H	Evu	Com	2718m;11^{0}36.776’N, 039^{0}13.814’E	MM144
100	*Pulicaria schimperi* DC.*	------	Asteraceae	H	Evu	Com	2483m; 11^{0}40.050’N, 039^{0}19.886’E	MM033
101	*Rhamnus prinoides* L’herit.	Gesho	Rhamnaceae	S	Hg	Com	2655m; 11^{0}40.161’N, 039^{0}19.811’E	MM064
102	*Rhus glutinosa* A.Rich. ssp. neoglutinosa^{+} M. Gilbert	Embis	Anacardiaceae	T	Al	Spa	2655m; 11^{0}40.161’N, 039^{0}19.811’E	MM065
103	*Rhus retinorrhoea* Oliv.	Tallo	Anacardiaceae	S	Ev	Rare	2193m; 11^{0}41.174’N, 039^{0}20.908’E	MM135
104	*Rhynchosia minima*.	Yems-jimat	Fabaceae	Cl	Fl	Com	2586m; 11^{0}40.177’N, 039^{0}19.960’E	MM160
105	*Ricinus communis* L.	Agullo	Euphorbiaceae	H	Hg	Com	2658m; 11^{0}40.171’N, 039^{0}19.812’E	MM056
106	*Rubus apetalus* Poir.	Enjory	Rosaceae	S	Ev	Rare	2281m; 11^{0}41.246’N, 039^{0}21.172’E	MM099
107	*Rumex abyssinicus* Jacq.	Embari-kolla	Polygonaceae	H	Fl	Spa	2905m; 11^{0}39.961’N, 039^{0}19.073’E	MM059
108	*Rumex nepalensis* Spreng.	Tult	Polygonaceae	H	Fl	Com	2675m; 11^{0}40.175’N, 039^{0}19.813’E	MM037
109	*Rumex nervosus* Vahl.	Embacho	Polygonaceae	S	Al	Com	2651m; 11^{0}38.705’N, 039^{0}18.956’E	MM007
110	*Ruta chalepensis* L.***	Tenadam	Rutaceae	H	Hg	Spa	2675m; 11^{0}40.175’N, 039^{0}19.813’E	MM038
111	*Salvia schimperi* Benth.	Gime-kitel	Lamiaceae	H	Evu	Com	2662m; 11^{0}40.177’N, 039^{0}19.813’E	MM9
112	*Schinus molle* L.***	Kundoberbere	Anacardiaceae	T	Hg	Spa	2738m; 11^{0}40.415’N, 039^{0}19.476’E	MM069
113	*Sesamum orientale* L.***	Selit	Pedaliaceae	H	Fl	Rare	2097m; 11^{0}38.259’N, 039^{0}20.309’E	MM072
114	*Sida schimperiana* Hochst.ex A.Rich.	Chifrig	Malvaceae	H	Evu	Com	2667m; 11^{0}40.357’N, 039^{0}20.009’E	MM153
115	*Silene macrosolen* A.Rich.	Wegert	Caryophyllaceae	H	Evu	Rare	2832m; 11^{0}40.231’N, 039^{0}19.202’E	MM62
116	*Solanecio gigas*$^{+}$ (Vatke.) C.Jeffrey	Shikokogomn	Asteraceae	S	Hg	Spa	2667m; 11^{0}40.604’N, 039^{0}20.817’E	MM159
117	*Solanum anguivi* Vatke.	Zirch Embay	Solanaceae	S	Evu	Rare	2661m; 11^{0}40.174’N, 039^{0}19.811’E	MM120
118	*Solanum incanum* L.	Embuay	Solanaceae	S	Evu	Com	2209m;11^{0}37.411’N,039^{0}19.239’E	MM008
119	*Solanum marginatum*$^{+}$ L.f.	Geber-embuay	Solanaceae	S	Evu	Spa	2832m; 11^{0}40.231’N, 039^{0}19.202’E	MM061
120	*Solanum nigrum* L.	Tikur Awut	Solanaceae	H	Al	Spa	2668m; 11^{0}38.200’N, 039^{0}19.877’E	MM026

121	*Stephania abyssinica* (Dillon & A.Rich.) Walp.	Yeayit-hareg	Menispermaceae	Cl	Evu	Com	2584m; 11^{0}38.128’N, 039^{0}18.496’E	MM123
122	*Striga hermonthica** (Del.) Benth.	Yemashila-Kitign	Scrophulariaceae	H	Fl	Rare	2524m; 11^{0}40.041’N, 039^{0}19.876’E	MM034
123	*Thymus schimperi*$^+$ *Ronniger*	Tosign	Lamiaceae	H	Evu	Com	3202m; 11^{0}40.520’N, 039^{0}16.534’E	MM105
124	*Tragia brevipes** Pax.	Ablalit	Euphorbiaceae	H	Evu	Rare	2098m; 11^{0}38.223’N, 039^{0}20.312’E	MM077
125	*Urtica simensis*$^+$ Steudel.	Sama	Urticaceae	H	Hgw	Spa	2584m; 11^{0}40.074’N, 039^{0}19.594’E	MM051
126	*Verbena officinalis* L.	Atuch	Verbenaceae	H	Fl	Rare	2268m; 11^{0}40.868’N, 039^{0}21.575’E	MM101
127	*Verbscum sinaiticum* Benth.	Ketetina	Scrophulariaceae	H	Al	Com	2655m; 11^{0}40.160’N, 039^{0}19.810’E	MM063
128	*Vernonia leopoldii*$^+$ (Sch. Bip.ex Walp.) Vatke	Kokemeri	Asteraceae	S	Evu	Com	2392m; 11^{0}38.818’N, 039^{0}19.454’E	MM046
129	*Vernonia schimperi* DC.	Yemich	Asteraceae	S	Evu	Com	2392m; 11^{0}38.818’N, 039^{0}19.454’E	MM20
130	*Vicia faba* L.***	Bakela	Fabaceae	H	Fl	Com	2659m; 11^{0}40.171’N, 039^{0}19.812’E	MM057
131	*Withania somnifera* (L.) Dunal in DC.	Gizewa	Solanaceae	S	Hgw	Rare	2651m; 11^{0}38.147’N, 039^{0}19.830’E	MM027
132	*Zehneria scabra* (Linn.f.) Sond.	Aregresa	Cucurbitaceae	Cl	Hgw	Com	2654m; 11^{0}38.141’N, 039^{0}19.828’E	MM24
133	*Zingiber officinale* Roscoe***	Zingibl	Zingebraceae	H	Hg	Rare	1892m; 11^{0}38.818’N, 039^{0}19.454’E	MM142
